# The Ebola Interferon Inhibiting Domains Attenuate and Dysregulate Cell-Mediated Immune Responses

**DOI:** 10.1371/journal.ppat.1006031

**Published:** 2016-12-08

**Authors:** Ndongala Michel Lubaki, Patrick Younan, Rodrigo I. Santos, Michelle Meyer, Mathieu Iampietro, Richard A. Koup, Alexander Bukreyev

**Affiliations:** 1 Department of Pathology, the University of Texas Medical Branch, Galveston, Texas, United States of America; 2 Galveston National Laboratory, the University of Texas Medical Branch, Galveston, Texas, United States of America; 3 Immunology Laboratory, Vaccine Research Center, National Institute of Allergy and Infectious Diseases, National Institutes of Health, Bethesda, Maryland, United States of America; 4 Department of Microbiology & Immunology, the University of Texas Medical Branch, Galveston, Texas, United States of America; St. Jude Children's Research Hospital, UNITED STATES

## Abstract

Ebola virus (EBOV) infections are characterized by deficient T-lymphocyte responses, T-lymphocyte apoptosis and lymphopenia. We previously showed that disabling of interferon-inhibiting domains (IIDs) in the VP24 and VP35 proteins effectively unblocks maturation of dendritic cells (DCs) and increases the secretion of cytokines and chemokines. Here, we investigated the role of IIDs in adaptive and innate cell-mediated responses using recombinant viruses carrying point mutations, which disabled IIDs in VP24 (EBOV/VP24m), VP35 (EBOV/VP35m) or both (EBOV/VP35m/VP24m). Peripheral blood mononuclear cells (PBMCs) from cytomegalovirus (CMV)-seropositive donors were inoculated with the panel of viruses and stimulated with CMV pp65 peptides. Disabling of the VP35 IID resulted in increased proliferation and higher percentages of CD4^+^ T cells secreting IFNγ and/or TNFα. To address the role of aberrant DC maturation in the IID-mediated suppression of T cell responses, CMV-stimulated DCs were infected with the panel of viruses and co-cultured with autologous T-lymphocytes. Infection with EBOV/VP35m infection resulted in a significant increase, as compared to wt EBOV, in proliferating CD4^+^ cells secreting IFNγ, TNFα and IL-2. Experiments with expanded CMV-specific T cells demonstrated their increased activation following co-cultivation with CMV-pulsed DCs pre-infected with EBOV/VP24m, EBOV/VP35m and EBOV/VP35m/VP24m, as compared to wt EBOV. Both IIDs were found to block phosphorylation of TCR complex-associated adaptors and downstream signaling molecules. Next, we examined the effects of IIDs on the function of B cells in infected PBMC. Infection with EBOV/VP35m and EBOV/VP35m/VP24m resulted in significant increases in the percentages of phenotypically distinct B-cell subsets and plasma cells, as compared to wt EBOV, suggesting inhibition of B cell function and differentiation by VP35 IID. Finally, infection with EBOV/VP35m increased activation of NK cells, as compared to wt EBOV. These results demonstrate a global suppression of cell-mediated responses by EBOV IIDs and identify the role of DCs in suppression of T-cell responses.

## Author Summary

The extensive investigation of interferon antagonism mediated by Ebola virus (EBOV) over the last 16 years resulted in identification of two interferon inhibiting domains (IIDs) located in the VP24 and VP35 proteins of the virus and of multiple mechanisms by which the domains disable the innate immune system and promote replication of the virus. However, the effects of these domains on cell-mediated immune response had not been investigated. To determine the effects of IIDs on cell-mediated responses, we used a panel of recombinant strains of EBOVs with point mutations disabling the VP24 and/or VP35 IIDs. The viruses were used for infection of peripheral blood mononuclear cells (PBMCs) or dendritic cells (DCs), which were subsequently co-cultured with T cells. We found that IIDs block activation and proliferation of T cells as a result of their functional role in suppressing maturation of DCs and limiting the formation of immunological synapses. Similarly, IIDs were demonstrated to suppress activation and differentiation of B cells, and skew activation of NK cells present in infected PBMCs. These data provide evidence of previously unknown effects of IIDs on the adaptive and innate cell-mediated immune responses and identify a novel mechanism of “immune paralysis” during EBOV infections.

## Introduction

The 2013–2016 outbreak of Ebola virus (EBOV) in West Africa claimed the lives of 11,300 people [1]. EBOV infections are characterized by ‘immune paralysis’, the profound immune deficiency resulting in uncontrolled viral replication [2]. A characteristic feature of EBOV infections is lymphopenia, which is observed in both humans and experimentally infected nonhuman primates (NHPs) [3–10] and is particularly pronounced during fatal human cases [9–11]. Fatal human cases and studies with EBOV-infected NHPs also demonstrated apoptosis of T cells accompanied by upregulation of tumor necrosis factor related apoptosis inducing ligand (TRAIL) and Fas/FasL [11, 12]. Moreover, EBOV infection of macaques resulted in depletion of T-cells, NK-cells but not CD20^+^ B cells, and no detectable activation of T-cell [4]. The lack of T cell activation in infected macaques contrasts a recent study of EBOV survivors, which received EBOV-specific antibody treatment and demonstrated a substantial immune activation of T and B cells [13]. Thus, the available information on the effect of EBOV on cell-mediated response is incomplete and controversial.

Type I interferons (IFN-I) are well-characterized inflammatory mediators whose interaction with IFNα/β receptors (IFNAR) is critical for controlling viral infections [reviewed in reference[14]. IFNAR induces the Janus activated kinase-signal transducer that results in activation of transcription JAK-STAT pathway in the majority of cells, along with other pathways, some of which are cell type-specific, which jointly transcriptionally control expression of hundreds of IFN-stimulated genes (ISG) [15]. IFN-I directly regulates activation of numerous immune cell types including dendritic cells (DCs), T-lymphocytes, B-lymphocytes and NK cells [16–22]. IFN-I has been shown to affect monocyte and macrophage functions and differentiation [14, 23, 24]. Furthermore, IFN-I stimulates antibody-dependent cytotoxicity of macrophages while exerting both positive and negative regulation of secreted inflammatory mediators [14, 25]. IFN-I triggers macrophages to upregulate nitric oxide synthase 2, resulting in enhanced IFNγ-induced oxidative response and eventually enhanced phagocytosis [17, 26, 27]. With regards to DCs, IFN-I has multiple effects, including differentiation from monocytes, maturation and migration [23, 28–32] and enhancing their antigen presentation capacity [14, 16, 23, 24, 28–30, 33]. The presence of IFN-I during antigen-dependent maturation of DCs has been shown to strongly enhance their capacity to induce human antibody responses and CTL expansion [18, 19, 34, 35]. IFNα/β stimulation of immature DCs leads to a rapid upregulation of cell surface markers associated with the initiation of an adaptive immune response including MHC class I, MHC class II, CD40, CD80, CD86 and CD83 [16, 24, 33]. INFα has been shown to promote expression of chemokine receptors such CC chemokine receptor type 7 (CCR7) while both IFNα and IFNβ are required for the migration of plasmacytoid DCs to the marginal zones occupied by T-lymphocytes *in vivo* [36].

Signaling through IFNAR in T-lymphocytes is critical to the development of their effector functions. As noted above, IFN-I increases presentation of MHC-associated antigenic peptides on the surface of antigen presenting cells (APCs) that in turn results in antigen-specific activation of T-lymphocytes. IFN-I can exert it effects on immune cells either directly, through IFNAR signaling, or indirectly by the induction of chemokines, which promote recruitment of immune cells to the site of infection and result in the release of a second wave of immune modulatory cytokines [36]. Studies in mice have shown that IFNα promotes efficient cross-priming of antigen-specific CD8^+^ T-cells and secretion of IFNγ [18, 37]. IFNα has been shown to be a critical regulator of genes involved in the CTL responses [38, 39]. IFN-I stimulation of naïve CD4^+^ T-cells results in their differentiation into IFNγ-producing Th1 cells [19]. IFN-I directly enhances the functional role of CD4^+^ T-cells in the development of antibody responses [20]. Furthermore, IFN-I have been shown to directly inhibit regulatory T-cell function thereby promoting optimal antiviral T-cell responses during acute infection [21]. Conversely, IFN-I, along with IFN-III, may directly suppress proliferation of CD4^+^ T cells in context of viral infection [40]. Hence, IFN-I exhibits complex, pleotropic effects that are T lymphocyte subset specific.

IFN-I also has a profound effect on immune cells other than DCs and T cells. In addition to the ability to enhance antibody responses through the effects on DC and CD4^+^ T cells mentioned above, IFN-I also directly stimulates the ability of B cells to secrete antibodies [20]. IFN-I is critically associated with the production of all subtypes of immunoglobulin G (IgG) and the development of long-lived plasma cells and immunological memory [20, 41–43]. IFN-I was shown to enhance secretion of IFNγ and numerous other cytokines by NK cells through an autocrine IFNγ-dependent activity and enhance cytolytic activity [22, 44]. IFN-I also promotes both expansion and survival of proliferating NK cells *via* IFN-I/STAT1-dependent production of IL-15 [14, 45, 46]. Due to these pleiotropic effects, viruses have evolved targeted subversion strategies aimed at blocking IFN-I signal transduction by targeting the JAK-STAT pathway.

One of the characteristic features of EBOV infection is the strong antagonism of IFN-I responses by IFN-inhibiting domains (IID) located in the viral proteins VP24 and VP35, including the suppression of cytosolic sensing of double stranded RNA by VP35 IID and the subversion of IFN-induced signaling by both VP35 and VP24 IIDs [reviewed in reference[47]. Another important feature of EBOV infections is the lack of maturation of DCs despite their susceptibility to the virus [48, 49]. We recently demonstrated that a point mutation disabling the VP35 IID effectively unblocks maturation of DCs exposed to the virus, while a mutation disabling VP24 IID promotes partial maturation [50]. These mutations result in a global modulation of infected DCs transcriptome profiles with only partial overlapping being observed; hence, VP24 and VP35 IIDs are associated with distinct antagonistic mechanisms [51].

In this study we attempted to determine the effects of EBOV VP24 and VP35 IIDs on global adaptive and innate cell-mediated responses. We used recombinant viruses carrying point mutations disabling IID in VP24 (EBOV/VP24m), VP35 (EBOV/VP35m) or both (EBOV/VP35m/VP24m) previously generated in our lab [50, 51] each expressing green fluorescent protein (GFP) to visualize the infection of susceptible cells. For comparisons, GFP-expressing virus with no mutations in IIDs [52] which otherwise is identical to the mutated viruses, referred here as wild type (wt) EBOV, was used. Many important findings on EBOV pathogenesis utilize a mouse model; however, IFN-I has different effects on human and mouse lymphocytes [53, 54]. We therefore used only primary human immune cells in these studies. We found that IIDs result in a global attenuation and dysregulation of cell-mediated responses, including T, B and NK cells.

## Results

### EBOV suppresses activation of T cells

**Fig 1:**
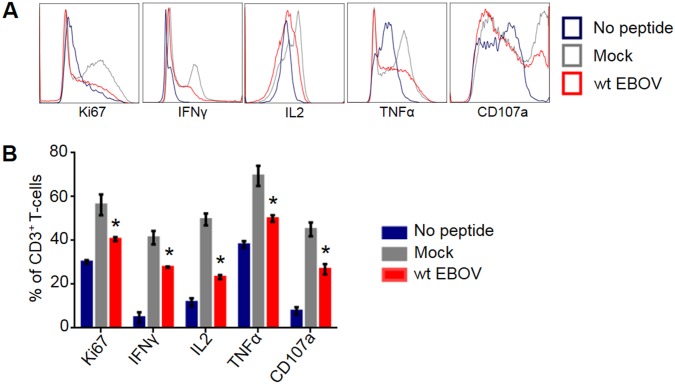
EBOV suppresses activation of T cells. CMV-specific T-cell responders were isolated and expanded from CMV^+^-donors as described in the Materials and Methods section. In parallel, monocytes were isolated and differentiated into DCs from autologous donors. DCs were simultaneously pulsed with CMV-peptides and infected with wt EBOV. Following overnight stimulation, expanded CMV-responders were added at a 1:1 ratio with control (no peptide) or CMV-pulsed DCs. **A.** Representative flow cytometry histograms showing the levels of the proliferation marker Ki67, cytokines associated with T-cell activation, and the marker of degranulation CD107a. **B.** Percentage of markers associated with activation in one representative donor. Mean values with SE based on triplicate wells from a single donor. The asterisks indicate statistically significant differences of wt EBOV-infected samples (p<0.05) compared to mock. Testing of T cells from another donor resulted in essentially similar data.

To determine the extent of EBOV-mediated suppression of cell-mediated immune response in our experimental systems, we assessed the ability of mock and EBOV-infected DCs to stimulate CMV-specific T-lymphocytes isolated from healthy individual donors seropositive for cytomegalovirus (CMV). CD14^+^ monocytes were isolated from peripheral blood mononuclear cells (PBMCs) using magnetic bead-based methods and differentiated into DCs for 7 days. Differentiated immature DCs were infected with wt EBOV expressing enhanced green fluorescent protein (GFP) from an added gene referred here as wild type (wt) EBOV [52] and simultaneously pulsed with pooled peptides overlapping CMV pp65 immunodominant protein followed by incubation for an additional 18–24 hours. In parallel, autologous PBMCs were stimulated with CMV peptides for 48 hours, at which point CMV-specific CD137^+^ T-lymphocytes were isolated and expanded for 8 days. Expanded responder cells were added at day 8 at a 1:1 ratio with mock or EBOV-infected DCs. Following overnight stimulation, cells were stained as described in the Materials and Methods and analyzed for markers of activation. Non-peptide stimulated DCs were used as a control to determine specific induction of CMV responders over background. A significant increase in percentages of T-lymphocytes positive for Ki67, a nuclear marker of proliferation [55], was detected as compared to no peptide control (Fig 1A). When T-lymphocyte responders were cultured in the presence of EBOV-infected DCs, percentages of Ki67^+^ cells were significantly reduced as compared to mock. Intracellular cytokine staining paralleled these findings as infection of DCs with EBOV significantly reduced percentages of T cells positive for activation markers IFNγ, IL2 or TNFα, as well as for the marker of degranulation CD107α^+^ [56] (Fig 1A and 1B). Despite a reduction in the proportion of activated T cells in response to EBOV infection of DCs, we also observed a moderate increase of the proportion of activated T cells over no peptide controls. This is likely due to the presence of DCs with the lack of high level EBOV replication, which are expected to display MHC-CMV peptides, as infection of DCs with EBOV results in different levels of viral replication in individual cells present in the population [50, 51]. Overall, these findings are highly correlative with previous *in vivo* data on the deficient T cell response to EBOV infection.

### The VP35 IID reduces the Th1 responses

**Fig 2:**
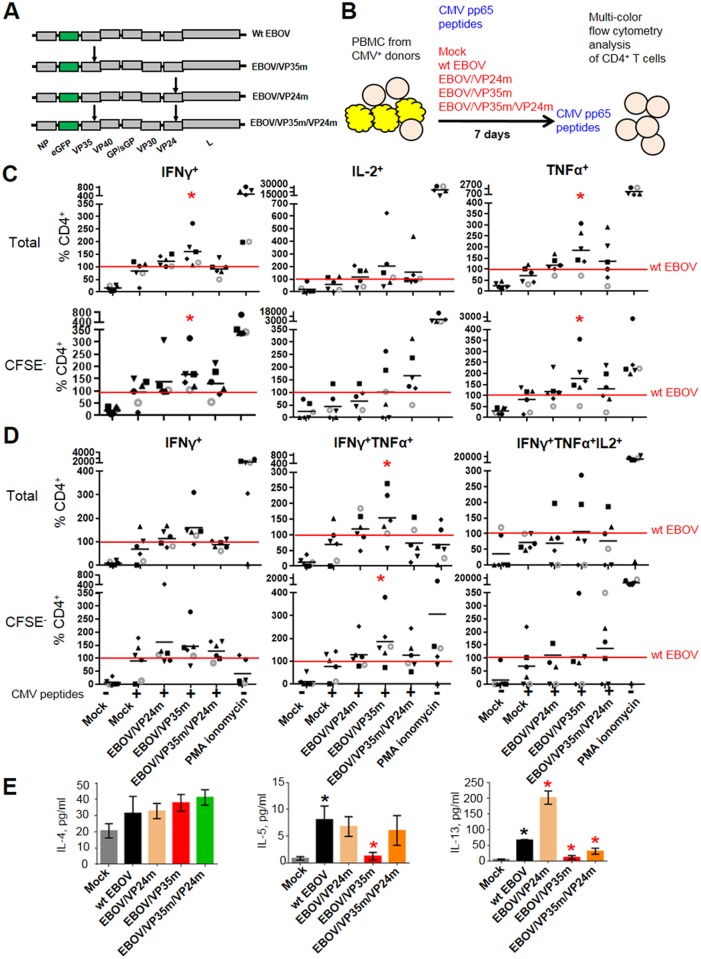
The VP35 IID reduces Th1 responses. **A.** Schematic representation of the genomes of wt EBOV and mutant EBOVs used to infect cells; mutations R312A in VP35 and K142A in VP24 are indicated by arrows, and the inserted GFP gene is indicated by green rectangles. **B.** Schematic representation of the cultivation experiment: PBMCs from CMV-positive donors were labeled with CFSE, inoculated with the indicated viruses at MOI of 2 PFU/cell or SEB, and stimulated with CMV pp65 peptides for 4 hours. PBMCs were washed and cultured for 7 days. Thereafter, cells were re-stimulated for 6 hours in culture medium containing CMV pp65 peptides in presence of brefeldin A, monensin, anti-CD28 mab, anti-CD49d mab and DNase. As a positive control, cells were stimulated with SEB and treated with phorbol-12-myristate-13 acetate (PMA) and ionomycin. Cells were stained intracellularly for the indicated cytokines followed by multicolor flow cytometry. **C.** Percentages of total and proliferating (CFSE^-^) CD4^+^ T cells secreting total IFNγ, IL-2 or TNFα normalized to wt EBOV (100%, indicated by the red horizontal lines). Percentages of proliferating and total CD4^+^ T cells were individually normalized to wt EBOV values in the same donor (100%, indicated by the red horizontal lines). Cells from each individual donor are represented by the same symbol across the panels; mean values are indicated by horizontal bars. Red asterisks indicate significant differences to wt EBOV (p<0.05). **D.** Analysis of total and proliferating (CFSE-) CD4^+^ T cells secreting combinations of multiple cytokines using Boolean gating: percentages of CD4^+^ T cells positive for the indicated cytokines and negative for the other cytokines included in the analysis; otherwise the designations are same as in Panel C. **E.** Concentrations of IL-4, IL-5 and IL-13 in supernatants of infected DCs co-cultured with expanded CMV-specific responder T-lymphocytes for 24 hours. Mean values with SE based on three donors. Black asterisks indicate significant differences to mock, and red asterisks indicate significant differences to wt EBOV infection (p<0.05). The experiment was performed 3 times with different donors, with essentially same results.

To determine the effects of IID on T cell response, we utilized a panel of recombinant EBOV mutants, each expressing GFP from an added gene to visualize the infection, which included EBOV/VP24m with the VP24 IID disabled by the mutation K124A [50], EBOV/VP35m with the VP35 IID disabled by the mutation R312A, and EBOV/VP35m/VP24m with both mutations [51] (Fig 2A). PBMCs from CMV-seropositive donors were infected with the panel of recombinant EBOVs and simultaneously stimulated with CMV pp65 peptides for 7 days, and then re-stimulated with the peptides for 6 hours (Fig 2B). Multi-color flow cytometry analysis of CD4^+^ T cells secreting IFNγ, IL-2 or TNFα as markers of activation demonstrated that disabling of the VP35 IID, but not the VP24 IID, resulted in significantly increased percentages of IFNγ^+^ and TNFα^+^ cells (Figs 2C and S1, S1 Table). The observed effects were more prominent in the dividing 5, 6-carboxyfluorescein diacetate succinimidyl ester (CFSE)-negative cell population. Since the functionality of T cells generally correlates with the number of cytokines and other markers of activation simultaneously expressed by individual cells [57, 58], we next quantified CD4^+^ T cells positive or negative for all 32 possible combinations of IFNγ, IL-2, IL-4, IL-17a or TNFα by Boolean gating. We found two abundant populations: IFNγ^+^ and IFNγ^+^TNFα^+^ (S2 Fig). Comparison of these populations and IFNγ^+^TNFα^+^IL-2^+^, expected to be the most highly activated, demonstrated that disabling of VP35 IID results in the increase of IFNγ^+^TNFα^+^ cell populations (Fig 2D, S2 Table). We also detected the increase in the percentages of single-positive IFNγ^+^ cells in the majority of donors, but the effect did not reach statistical significance. Disabling of any of the two IIDs did not significantly affect the percentage of GFP^+^ cells, although some reduction was observed when both IIDs were disabled (S3A Fig). These data suggest that the effects of mutations on activation of T cells are not related to changes in viral replication. We next determined the levels of IL-4, IL-5 and IL-13, which are markers of Th2 response, in supernatants. Wt EBOV strongly induced the expression of IL-5 and IL-13 (but not IL-4), while disabling of the VP35 IID resulted in their significant reduction (Fig 2E). These findings suggest that VP35 IID strongly reduces Th1 response.

### The VP35 IID-mediated reduction of Th1 response results from the deficient maturation of DC

**Fig 3:**
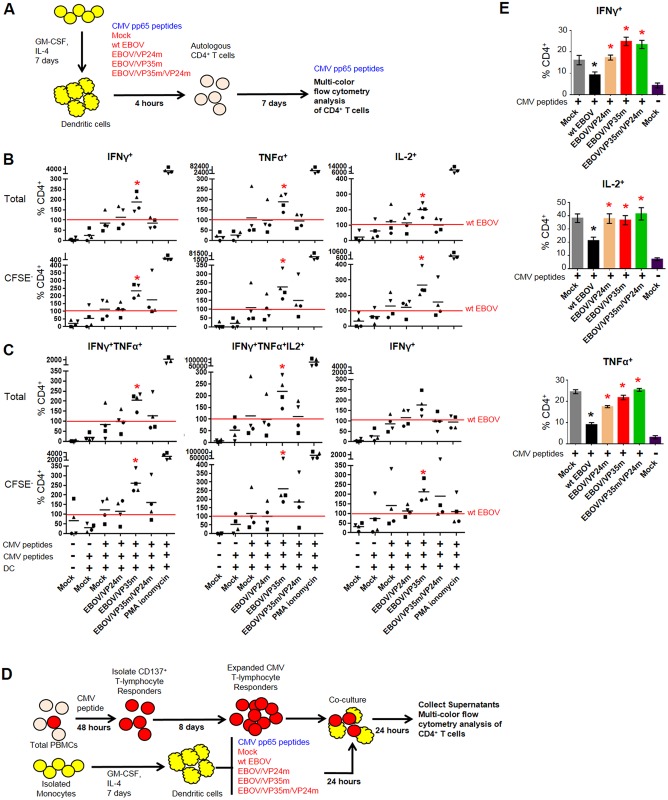
The VP35-mediated reduction of Th1 response results from the deficient maturation of DC. **A.** Schematic representation of the cultivation experiment: immature DCs from CMV-positive donors were inoculated with the indicated viruses and stimulated with CMV pp65 peptides for 4 hours, washed and co-cultured with CFSE-labeled autologous purified CD4^+^ T cells for 7 days. Thereafter, cells were processed and analyzed as described in the legend for Fig 2B. **B.** Percentages of total and proliferating (CFSE^-^) CD4^+^ T cells secreting total IFNγ, IL-2 or TNFα individually normalized to wt EBOV in the same donor; see legend for Fig 2C for details. **C**. Analysis of total and proliferating (CFSE-) CD4^+^ T cells secreting combinations of multiple cytokines using Boolean gating; see legend for Fig 2D for details. **D.** Schematic of T-lymphocyte responders assay. PBMCs were stimulated with CMV peptides for 48-hours and used for positive selection of CD137^+^ T-lymphocyte. Isolated CMV responders were expanded using dynabeads human T-cell activator beads for 8 days. In parallel, positively selected CD14^+^ monocyte were differentiated into DC for 7 days, stimulated with CMV peptides and infected with the panel of viruses. After 24 hours, expanded CMV-responders were added to stimulated/infected DCs. Following a 24 hour co-culture, supernatants were collected and cells were analyzed by flow cytometry. **E.** Percentages of CD4^+^ T-cells staining positive for intracellular IFNγ, IL-2 and TNFα determined by flow cytometry following a 24 hour co-culture of expanded CMV-specific T-lymphocyte responders with CMV-stimulated DCs infected with the indicated viruses. Black asterisks indicate significant differences to mock, and red asterisks indicate significant differences to wt EBOV infection (p<0.05). The experiment was performed 3 times with different donors, with essentially same results.

Since T cells are resistant to EBOV infection [3], mechanisms associated with the VP35 IID-induced suppression of the Th1 response may be related to infection of multiple non-lymphoid immune cells susceptible to EBOV. We hypothesized that the effect results from the deficient maturation of DCs associated with VP35 IID [50]. To test the hypothesis, we established a co-cultivation system, which included monocyte-derived DCs from CMV-positive donors, which were infected with the panel of viruses in the presence of CMV pp65 peptides and subsequently cultured with autologous purified CD4^+^ T cells (Fig 3A). Following 7 days of co-cultivation, cells were re-stimulated with CMV peptides for 6 hours, and CD4^+^ T cells were analyzed by multi-color flow cytometry. We found that disabling the VP35 IID strongly increased the proportion of total CD4^+^ T cells populations secreting IFNγ, TNFα or IL-2 (Figs 3B and S4, S3 Table). The effect was more pronounced in the actively dividing CFSE^-^ population. Once again, disabling of any of the two IIDs did not significantly affect the percentage of GFP^+^ cells, and a small reduction was observed with both IIDs disabled (S3B Fig), suggesting the effects of the mutations are not due to changes in the viral replication. To check if the time of peptide stimulation affects the observed effects, we compared simultaneous infection and stimulation used in our experiments, with stimulation 24 hours after infection. We found that the time of stimulation did not affect the percentage of GFP^+^ DCs (S5A Fig) or the percentage of activated IFNγ^+^ CD4^+^ T cells (S5B Fig).

**Fig 4:**
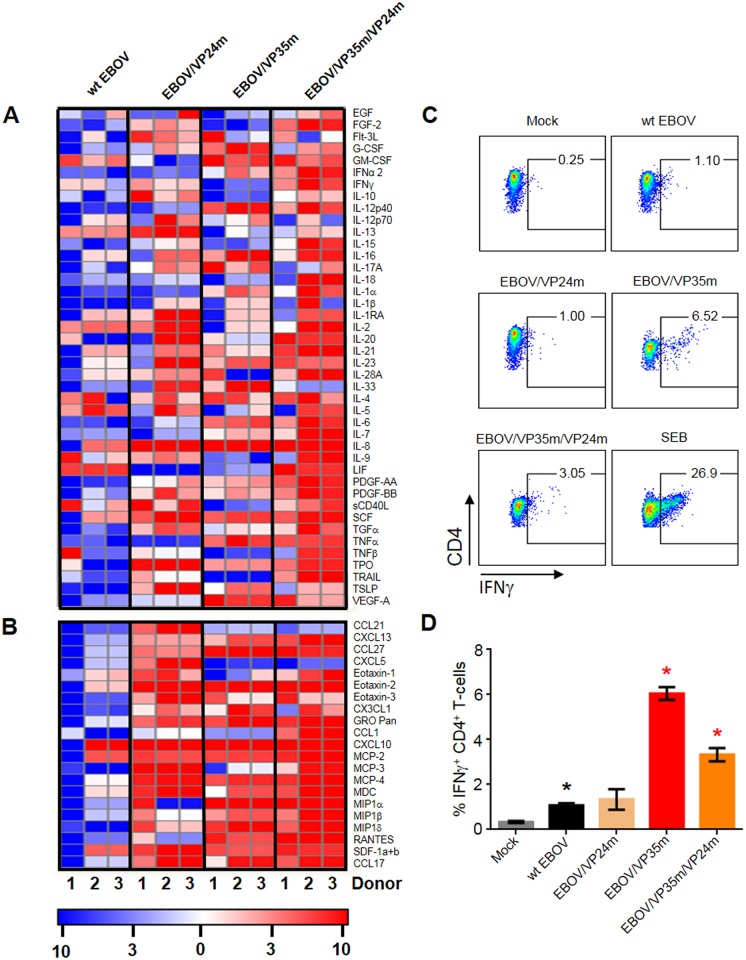
Disabling of IIDs changes cytokine and chemokine expression in co-cultures of CD4^+^ T cells with infected DCs. **A, B.** CMV-pulsed, EBOV-infected DCs were co-cultured with expanded CMV-specific responder T-lymphocytes for 24 hours, and concentrations of cytokines (**A**) and chemokines (**B**) in supernatants were determined using a bead-based multiplex analysis. Heatmap showing the relative fold change following normalization to mock-infected samples. Results from 3 individual donors are shown. **C**, **D.** Flow cytometry analysis of IFNγ^+^ CD4^+^ T cells in DC-T cell co-cultures after transfer of conditioned media from DCs infected with the indicated viruses: representative primary data (**C**) and mean values with SE based on triplicate samples (**D**). Black asterisks indicate significant differences to mock, and red asterisks indicate significant differences to wt EBOV infection (p<0.05). The experiment was performed two times with different donors, with essentially same results.

We next quantified CD4^+^ T cells positive or negative for IFNγ, IL-2, IL-4, IL-17a and TNFα by Boolean gating, resulting in 32 possible combinations. The vast majority of cells exposed to the panel of viruses or SEB belonged to the IFNγ^+^, TNFα^+^ and IL-4^+^ single-positive populations, and IFNγ^+^TNFα^+^ double-positive populations (Figs 3C, S6 and S7, S4 Table). Disabling of VP35 IID resulted in a significantly increased percentages of IFNγ^+^, IFNγ^+^TNFα^+^ and IFNγ^+^TNFα^+^IL-2^+^ cells. The increases were observed both in the dividing (CFSE^-^) and the total cell populations. To determine the effects of IID under conditions when the majority of CD4^+^ T cells respond to stimulation, CMV-specific CD4^+^ T cells from three donors were expanded and used in co-culture assays with autologous DCs (Fig 3D). Under these conditions, not only the disabling of VP35 IID, but also VP24 IID resulted in significant increases of cytokine secreting T cells compared to wt EBOV (Fig 3E). We next quantitated cytokines and chemokines in the medium of expanded CD4^+^ T cell responders cultured for 24 hours with CMV-pulsed DCs pre-infected with the panel of viruses (Fig 4, S5 Table). Infection of DCs with wt EBOV resulted in a significant reduction of the majority of cytokines and chemokines analyzed in comparison to mock-infected DCs, with a few exceptions, including IL-4, IL-5 and IL-13 (Fig 4A). These data further confirm suppression of T cell responses in general, and Th1 responses, by EBOV demonstrated in Figs 1 and 2. Disabling of either VP24 IID or VP35 IID resulted in an increase, as compared to mock-treated cells, of the majority of the cytokines analyzed in donors 2 and 3, and a lesser number of cytokines in donor 1. The effects of the two IIDs were not identical; disabling of the both of them increased almost all cytokines analyzed in donors 2 and 3, and approximately half in donor 1. Remarkably, the levels of IL-4, IL-5 and IL-13 did not increase compared to wt EBOV-infected DC. The effects of the mutations on chemokine expression was even stronger as the majority of those analyzed were upregulated in response to either or both mutations (Fig 4B).

We next determined if the induction of Th1 response associated with disabling of VP35 IID is related to soluble factors, by assessing the ability of conditioned media to activate naïve CD4^+^ T-cells. We therefore infected DCs with the panel of viruses or mock-infected and pulsed with CMV-peptides, incubated for 5 days, collected cell-free supernatants and transferred them to naïve CD4^+^ T cells. Following overnight stimulation, brefeldin A and monenesin were added to media for an additional 6 hours that was followed by intracellular staining of IFNγ. Culture of CD4^+^ T-cells in conditioned media from DCs infected with EBOV/VP35m or EBOV/VP35m/VP24m resulted in significantly increased percentages of IFNγ^+^ CD4^+^ T cells (Fig 4C and 4D). To further demonstrate the direct role of VP35 IID in the restriction of a Th1-response, lentiviral vectors expressing wild type or mutant VP35 were used to transduce DCs. Co-culture of CD4^+^ T-cells in the presence of DCs transduced with the mutant VP35 resulted in a significant increase in the percentages of IFNγ^+^ CD4^+^ T-cells compared to wild type VP35 (S8 Fig). Similar results were obtained both in the presence or absence of CMV peptides. These data suggest that the restriction of Th1 response by VP35 IID at least in part is mediated by soluble factors, and the observed effects are not related to changes in biological properties of the virus due to the introduced mutation. Taken together, the results demonstrate that the VP35 IID, and in a lesser degree VP24 IID, suppress activation of CD4^+^ T cells as a result of the IID-associated deficient maturation of DC.

### The suppressive effect of IID on DC maturation does not significantly result from the suppression of IFN-I signaling or of the released IFN

**Fig 5:**
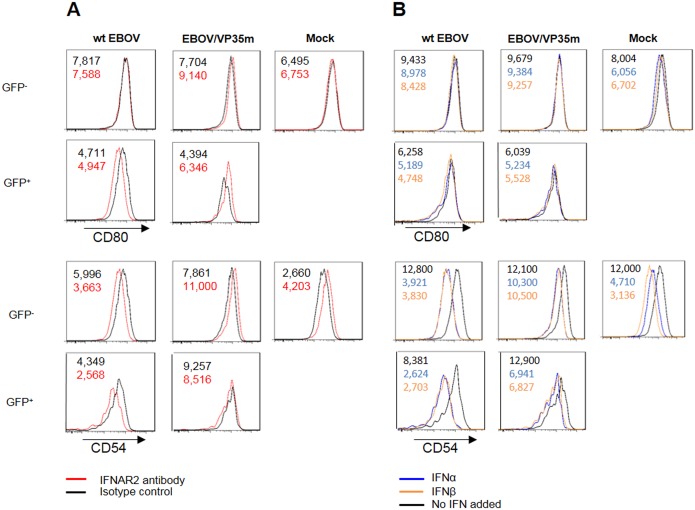
Suppression of IFN-I signaling and the prevention of IFN-I release by EBOV play only a limited role in inhibition of DC maturation. **A.** Effect of IFNAR2 blockade on expression of CD86. **B.** Effect of exogenously added IFNα and IFNβ on expression of CD86. Mean fluorescent intensity (MFI) for CD86^+^ mock-treated DC (black), or DC treated with IFNAR2 antibodies, IFNα, or IFNβ (colors) are indicated in the top left corners. The experiment was performed two times with different donors, with essentially similar results.

Previous reports have indicated that DCs require IFN-I response to mature [59]; however, induction of the IFN-I signaling pathway, but not release of IFN was reported to be a requirement for maturation of DCs following induction by negative-strand RNA viruses [60]. We therefore determined whether the observed suppression of DCs maturation by VP35 IID is a consequence of the suppression of IFN-I signaling and whether released IFN has any effect on this phenotype. First, we blocked IFN receptor 2 (IFNAR2), since the anti-viral activities of IFN-I correlate well with its binding to IFNAR2, rather than IFNAR-I [61]. For this purpose, IFNAR2 blocking antibodies were added to DCs at a concentration 30 μg/ml; we previously demonstrated that a 1,000-fold lesser dose of the antibody suppresses the expression of the IFN-inducible genes Mx1 and ISG56 in T cells [62]. Cells were incubated with IFNAR2 blocking antibodies at 37°C for 1 hour, followed by inoculation with the viruses as indicated previously. At 40 hours post infection, we examined the expression levels of markers associated with DC maturation including CD86, CD80 and CD54 (Figs 5A and S9A). Even though wt EBOV only induced low expression of CD86, IFNAR2 blockade further reduced it to the level of mock-infected DCs. No significant effects of the blockade were detected for CD80, and the effect on CD54 was similar to that of CD86, but somewhat less pronounced. The reduction of the level of CD86 in DCs infected with wt EBOV by the IFNAR2 blockade is likely a result of the synergistic effect of the blockade with the effects of the VP35 and VP24 IID present in the virus. In contrast, in DCs infected with EBOV/VP35m, the blockade did not result in significant changes in the expression of any of the three markers of maturation in both GFP^+^ and GFP^-^ cells. Since EBOV/VP35m has the intact VP24 IID, which inhibits IFN-I signaling, and VP35 IID suppresses not only double stranded RNA cytosolic sensing but also inhibits other host defense pathways [reviewed in reference[47], these findings suggest that DC maturation associated with disabling of VP35 IID is only partially dependent on IFN-I. This hypothesis is supported by the release of numerous cytokines including TNFα following infection with EBOV/VP35m (Fig 4A), which could stimulate DC maturation by induction of pathways other than the IFN-I pathway. Furthermore, these findings are consistent with the increased Th1 response by conditioned media from DCs infected with EBOV/VP35m (Fig 4C and 4D) and with the lack of difference between maturation of infected (GFP^+^) and uninfected (GFP^-^) DCs exposed to EBOV/VP35m we reported earlier [50]. These results differ from the previous observation with an HIV gag vaccine in mice, when blocking of IFN-I receptors prevented effective DCs maturation [59].

In separate experiments, the effects of exogenously added IFNα2 or IFNβ were determined. Due to the anti-viral effects of IFNs, DCs were infected and incubated for 24 hours before IFNα2 or IFNβ was added at the concentrations 1,000 and 800 IU, respectively. This was followed by an additional 20 hour-long incubation, and analysis of the expression of the maturation markers CD86 (Fig 5B), CD80, and CD54 (S9B Fig). No significant effect of exogenous IFN on the expression of CD86 and CD80 was found. Contrary to the expectations that exogenous IFNs would facilitate DCs maturation, the expression of CD54 was reduced, rather than increased in both the infected and mock-infected cells. In addition, no significant differences in the expression of the three maturation markers were found between GFP^+^ and GFP^-^ cells. These results support the previously published observation that secreted IFN-I is irrelevant for the induction of DC maturation by viruses [60]. Taken together, our results suggest that the suppression of IFN-I signaling and the prevention of IFN-I release by EBOV *per se* play only a limited role in the suppression of DCs maturation by the IID that is consistent with their diverse and highly redundant mechanisms of the suppression of the innate immune response by EBOV IIDs [47].

### The VP35 IID-mediated reduction of Th1 response is associated with impaired immunological synapses

**Fig 6:**
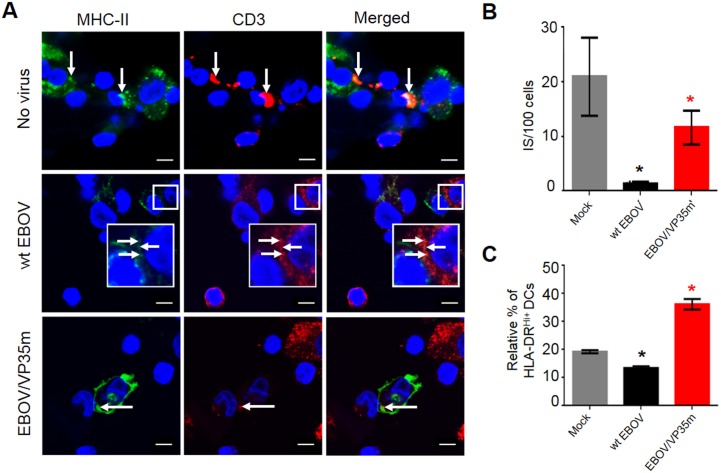
VP35 IID impairs the formation of immunological synapses. CMV-pulsed DCs infected with wt EBOV or EBOV/VP35m incubated with autologous CD4^+^ T cells. **A**. Confocal microscopy data showing co-localization of DC-expressed HLA-DR (green) and CD4^+^ T cell-expressed CD3 (red). Scale bar = 1 μm. Immunological synapses are shown by white arrows. Representative images from one of three donors analyzed. Note, a representative image of CD4^+^ T-cells co-cultured with wt EBOV infected DCs demonstrates one of a very limited number of dimly fluorescent HLA-DR^+^ cells co-localized with CD3^+^ T cells. Since HLA-DR staining of DCs infected with wt EBOV is dim, an inlet with increased brightness to visualize HLA-DR is shown. **B**. Numbers of CD4^+^ T cell—DC immunological synapses counted in 13 mm diameter cover slips. Results are expressed as numbers of immunological synapses per 100 cells. **C**. Percentages of HLA-DR^+^ DCs in infected or mock-infected DCs determined by flow cytometry. Mean values with SE based on triplicate samples from one of two independent experiments performed with different donors, which resulted in essentially same results. B, C, black asterisks indicate significant differences to mock, and red asterisks indicate significant differences to wt EBOV infection (p<0.05).

Stimulation of T cells by DCs is a key step, which affects the magnitude of the immune response to a viral infection [63]. To determine if VP35 IID interferes with the ability to form immunological synapses between DCs and T-lymphocytes, we co-cultured purified CD4^+^ T-cells with autologous CMV-pulsed DCs infected with wt EBOV or EBOV/VP35m. Twenty four hours following infection, autologous CD4^+^ T-cells were added at a 1:1 ratio, cultured for an additional 4 hours, fixed, and stained as described in the Materials and Methods. Immunological synapses between DCs and T cells were visualized by confocal microscopy, which demonstrated co-localization of HLA-DR and CD3ε in mock-infected cultures. Infection of DCs with wt EBOV resulted in a significantly reduced number of immunological synapses as compared to mock-infected DCs, while disabling of VP35 IID significantly increased the number in comparison to wt EBOV (Fig 6A and 6B). In addition, the intensity of the staining of the immunological synapses and their sizes were greater in DCs infected by EBOV/VP35m than in cells infected with wt EBOV. Furthermore, we noted that infection with wt EBOV reduced the HLA-DR expression compared to mock-infected cells, while no reduction was observed with EBOV/VP35m, the finding further confirmed by flow cytometry analysis (Fig 6C). Taken together these data suggest that EBOV VP35 IID interferes with IS formation by reducing DC maturation, thereby limiting the capacity of DCs to effectively generate an adaptive immune response.

### The VP35 and VP24 IID reduce T cell proliferation

**Fig 7:**
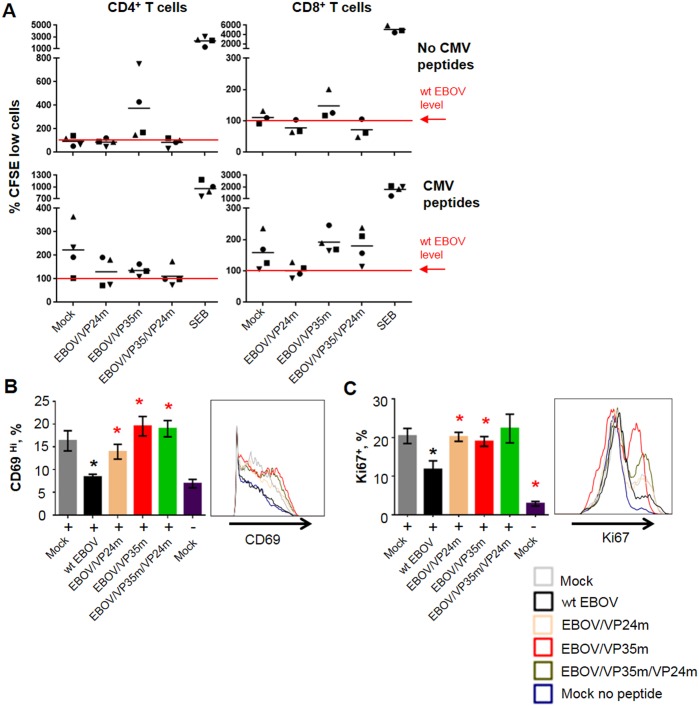
Effects of the VP35 and VP24 IID on proliferation of T cells. **A**, Proliferated CD4^+^ and CD8^+^ T cells analyzed by CFSE dilution assay, percentages of CFSE^low^ cells. Data for the mutated viruses are individually normalized to wt EBOV-infected cell levels in the same donor. Values for each of the 4 donors analyzed are indicated with individual symbols. Mean values for each treatment are indicated by horizontal bars. **B**, **C**. Expanded CD4^+^ T cell CMV-responders cultured with autologous CMV pulsed DCs: % of CD69^Hi^ cells (B) and Ki67 ^Hi^ CD4^+^ T cells (C) with representative primary data. Mean values with SE based on 3 donors/experiments. A, No statistically significant differences, B, C, statistically significant differences (P<0.05) for wt EBOV as compared to mock are indicated with black asterisks, and for the mutated viruses as compared to wt EBOV with red asterisks.

We next examined the effects of VP24 and VP35 IID on proliferation of T cells. Total PBMC from four healthy CMV-seropositive donors were labeled with CFSE, inoculated with the panel of viruses with or without simultaneous pulsing with CMV peptides, and cultured for 7 days (Figs 7A and S10, S6 Table). Infection with wt EBOV did not affect the percentages of the dividing CFSE^low^ cells without peptide stimulation, but reduced it, compared to uninfected cells, in presence of peptides. Infection with EBOV/VP35m resulted in an increase, as compared to wt EBOV, in proliferation of both CD4^+^ and CD8^+^ T cells in PBMCs from each donor, with or without peptides, although the effect was weak for CD4^+^ T-cells with peptides, and the overall effect did not reach statistical significance due to the high donor-to-donor variability. In contrast, no consistent effect was observed in PBMCs infected with EBOV/VP24m or EBOV/VP35m/VP24m.

Based on these data, we hypothesized that IID also suppresses expression of CD69, which is involved in lymphocyte proliferation as a signal transmitting receptor [64], and Ki67, a nuclear marker of proliferation. We utilized the enriched CMV responder assay described in Fig 3D, in which DCs and autologous isolated CMV-specific CD137^+^ CD4^+^ T-lymphocytes were cultured for 8 days, infected with the panel of the viruses, and pulsed with CMV peptides. Next, expanded CMV-specific T-lymphocyte responders were combined at a 1:1 ratio with the infected DCs and after 24 hours long co-cultivation, analyzed by flow cytometry. In T-cells cultured with wt EBOV infected DCs, the percentages of both CD69^+^ and Ki67^+^ CD4^+^ T cells were reduced as compared to T-cells cultured with uninfected DCs (Fig 7B and 7C) that suggests suppression of T cell activation/proliferation by EBOV and is consistent with the data shown in Figs 1 and 7A. Again, disabling of VP35 IID completely reversed the suppressive effect of wt EBOV. Unexpectedly disabling of VP24 IID, in addition to VP35 IID, or both, also completely reversed the suppressing effect of IIDs in this system. Taken together, these data demonstrate the suppressive effect of both VP35 and VP24 IID on T cell proliferation.

### VP35 and VP24 IID block phosphorylation of TCR complex-associated adapters and downstream signal molecules

**Fig 8:**
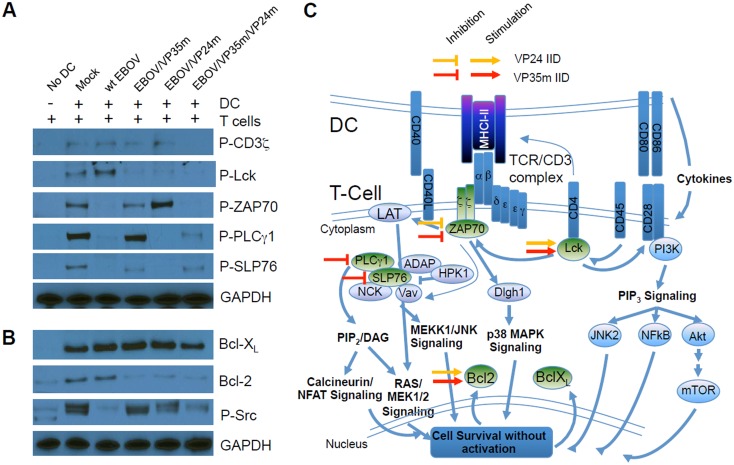
VP35 and VP24 IID block phosphorylation of TCR complex-associated adapters and downstream signal molecules. CD4^+^ T cells were co-cultured with DCs infected with wt EBOV or the mutated viruses for 4 days, and TCR signal transduction was analyzed by Western blotting of phosphorylated adapter molecules and downstream signaling molecules (**A**) and anti-apoptotic Bcl-2 family members and the SRC molecule (**B**). **C**. Map of signaling cascades associated with MHC-TCR engagement that promotes transduction of survival signals in the absence of antigen-dependent proliferation according reference [95]; the map itself is adapted from Cell Signaling Technology web site (http://www.cellsignal.com). As shown in panel A, basal phosphorylation of adapter molecules was observed following culture of autologous CD4^+^ T-cells with mock-DCs. The identified VP24 and VP35 IID-mediated blocks and stimulations of signal transduction are shown; note that block of signal transduction is associated with altered DCs function.

Previous studies have demonstrated that DCs and other APCs are vital to the survival of CD4^+^ T-cells due to MHC-TCR complex-dependent signal transduction that require a direct cellular contact [65–67]. We therefore sought to examine the effects of IID on signal transduction using co-cultures of unstimulated DCs and autologous CD4^+^ T-cells. Following a four-day co-culture with mock or EBOV-infected DCs, the phosphorylation cascades associated with TCR signaling were analyzed by Western blotting (Fig 8A). Specifically, we examined phosphorylation of TCR complex-associated adapters and downstream signal molecules, which have previously been shown to remain phosphorylated in the absence of antigen-dependent activation, and whose phosphorylation depends on DC-T-cell contact [65, 68–70]. In the absence of DCs, a limited phosphorylation of signal transduction mediators was detected in CD4^+^ T-cells. In contrast, CD4^+^ T-cells cultured in the presence of mock-infected DCs exhibited phosphorylation of molecules activated at both early and late stages of TCR-mediated signaling. Presumably, the observed phosphorylation status represents basal phosphorylation events that while supporting survival of CD4^+^ T-cells, remain below the threshold required for activation. Infection with wt EBOV resulted in the increase in phosphorylation of Lck; however, phosphorylation of additional adapters including ZAP70, PLCγ1 and SLP76 appeared to be blocked. Consistent with the data demonstrating reduced formation of immunological synapse (Fig 6), the absence of downstream phosphorylation events may be the result of impaired synaptic formation and/or aberrant signal transduction. Phosphorylation of ZAP70 is dependent on the formation of TCR microclusters that form scaffolding for downstream signaling events at the intracellular sites of immunological synapse formations [71]. Thus the absence of phosphorylated ZAP70 in co-culture of T-cells with wt EBOV-infected DCs strongly indicates at suboptimal engagement of TCR. In agreement with that, infection of DCs with wt EBOV significantly reduced expression of HLA-DR (Fig 6C). It is not immediately clear why a relatively strong induction of Lck phosporylation was observed following wt EBOV infection; however, it is plausible that secondary signal transduction may be suboptimal resulting in impaired downstream signaling.

Disabling of VP35 IID unblocked phosphorylation of ZAP70 and PLCγ1, and to a lesser degree, SLP76, while disabling of VP24 IID unblocked only ZAP70, but to a much greater degree than disabling of VP35 IID. Unexpectedly, infection with EBOV/VP24m resulted in an increase in the relative amount of phosphorylated ZAP70 despite the lack of phosphorylation of the downstream signaling molecules PLCγ1 and SLP76; however, this is consistent with the lack of effective T cell activation by this mutant. This discrepancy may result from differential kinetics associated with altered signaling events, the absence of co-activating signals or upregulation of negative regulators of PLCγ1 and SLP76 or other inhibitory factors downstream ZAP70. Surprisingly, disabling of both IIDs resulted in only weak phosphorylation of PLCγ1 and SLP76, but not ZAP70. We note however, the absence of CD3ζ and Lck phosphorylation in EBOV/VP35m/VP24m co-cultures, suggesting that the phosphorylation of PLCγ1 and SLP76 may be associated with alternate signaling pathways. Although not examined in these studies, it is possible that both phosphorylation and dephosphorylation kinetics may be variable following co-culture.

To further characterize the capacity of infected DCs to transmit survival signals, we determined the relative levels of the anti-apoptotic Bcl-2 family members. These proteins are involved in the survival of T cells and are upregulated in response to survival signals [72–74]; on the other hand, survival signal transduction was previously associated with reduced Bcl-2 levels [75–77]. We also determined phosphorylation of Src, which has previously been shown to be essential for naïve T-cell survival and also TCR-dependent [78] (Fig 8B). Bcl-X_L_ was undetectable in control CD4^+^ T-cells alone, but was readily detectable at relatively similar levels in CD4^+^ T-cells cultured with mock, wt or mutant EBOV infected DCs. Bcl-2 was readily detectable in CD4^+^ T-cells cultured with mock or wt EBOV-infected DCs, but greatly reduced when VP35 and/or VP24 IID were mutated. Furthermore, the phosphorylated Src was detectable in T cells cultured with mock-infected DCs and DCs infected with the mutated but not wt EBOV, showing correlation with activation of infected DCs (Figs 2–7). Taken together, these data demonstrate that cultivation of T cells with EBOV-infected DCs blocks phosphorylation of ZAP70, PLCγ1 and SLP76 involved in TCR signaling and the pro-survival molecule P-Src, and that the levels of P-Src correlate with T cell activation, while that of Bcl-2 correlate with T cell survival in the autologous system. Furthermore, these data identify the role of VP35 IID in blocking phosphorylation of these molecules, and role of VP24 IID in blocking phosphorylation of ZAP70. Paradoxically, the two IIDs demonstrated an opposite (i.e. stimulating) effect on phosphorylation of Lck, as well as on expression of the prosurvival molecule Bcl-2. A schematic which illustrates the positive and negative signals associated with IIDs as they relate to the observed phosphorylation status of adapter molecules in Fig 8A and 8B is presented in Fig 8C.

### The VP35 IID inhibits B-cell function and differentiation

**Fig 9:**
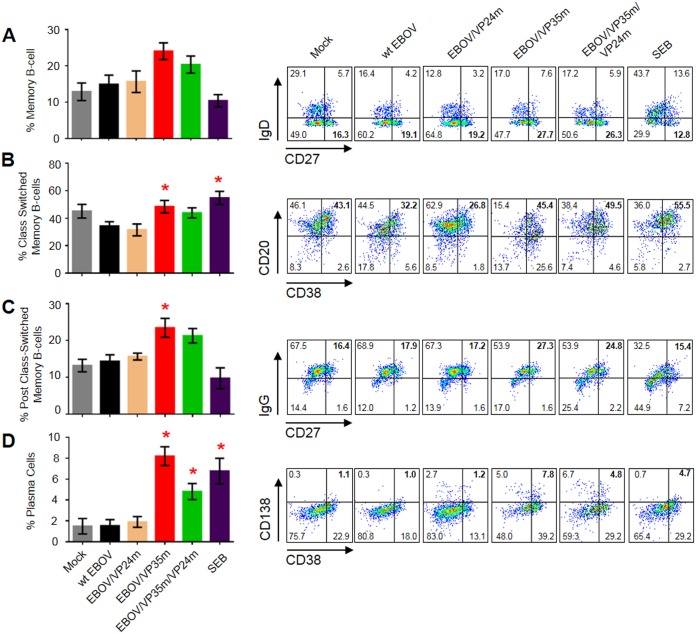
VP35 IID inhibits B-cell function and differentiation. Percentages of CD19^+^CD27^+^IgD^-^ memory B-cells (**A**), CD19^+^CD27^-^IgD^-^IgM^-^CD20^-^CD38^++^ class switched memory B-cells (**B**), CD19^+^CD27^+/Dim^IgD^-^IgM^-^CD20^-^CD38^-^ post class-switched memory B-cells (**C**), and CD19^+^CD38^+^CD138^+^ plasma cells (**D**) in PBMCs infected with wt EBOV or the mutant viruses. The subgating strategies are shown in S11B and S11C Fig. Average values based on 3 donors with SE shown. Statistically significant differences (P<0.05) for the mutated viruses as compared to wt EBOV are shown with red asterisks. Flow plots are representative primary data from one donor. Percentages of cell populations are indicated in each quadrant with that used for graphs at the left are bolded.

To date, only limited data concerning the effects of EBOV infection on B and NK cell function have been published. To determine the effects of EBOV infection and IID on B-cells, PBMCs were infected with wt or mutated EBOVs at MOI 1.0 PFU/cell, incubated for 7 days, and analyzed for markers of activation, maturation and those associated with specific subsets. The percentages of naïve B-cells (CD19^+^CD27^-^IgD^+^) were reduced after infection with wt EBOV or the mutants except the double mutant (S11A Fig). The percentages of memory B-cells (CD19^+^CD27^+^IgD^-^) did not change after infection with wt EBOV, while disabling of VP35 (but not VP24) IID increased this population (Fig 9A). Of note, these results inversely correlated with observed changes in the percentage of naïve B-cell populations (S11A Fig). We next examined the effects of IID on the percentages of class-switched memory B-cells (CD19^+^CD27^-^IgD^-^IgM^-^CD20^+^CD38^++^) (Figs 9B, S11B and S11C). Infection with wt EBOV slightly reduced the percentages of this population, while disabling of VP35 IID significantly increased it. Examination of post-class switched memory B-cells (CD19^+^CD27^+/Dim^IgD^-^IgM^-^CD20^-^CD38^-^) revealed a similar effect (Fig 9C); again, the decrease in SEB-treated cells is consistent with an increase in other subsets in samples treated with SEB (Fig 9B and 9D). These results suggest that VP35 IID may, by a yet to be determined mechanism, block the development of post-class switched memory cells. Analysis of plasma cells (CD19^+^CD38^+^CD138^+^) demonstrated the lack of effect of wt EBOV, but as much as 4-fold increase in their percentage after infection with EBOV/VP35m (Fig 9D). Interestingly, infection with the double mutant also increased the percentages of this cell population, but only ~1.5-fold, suggesting that VP24 IID reduces the effect of VP35 IID. These data demonstrate that VP35 IID suppresses induction of memory B cells, their class switching, and their differentiation into plasma cells.

### The VP35 and VP24 IIDs suppress activation of NK cells

**Fig 10:**
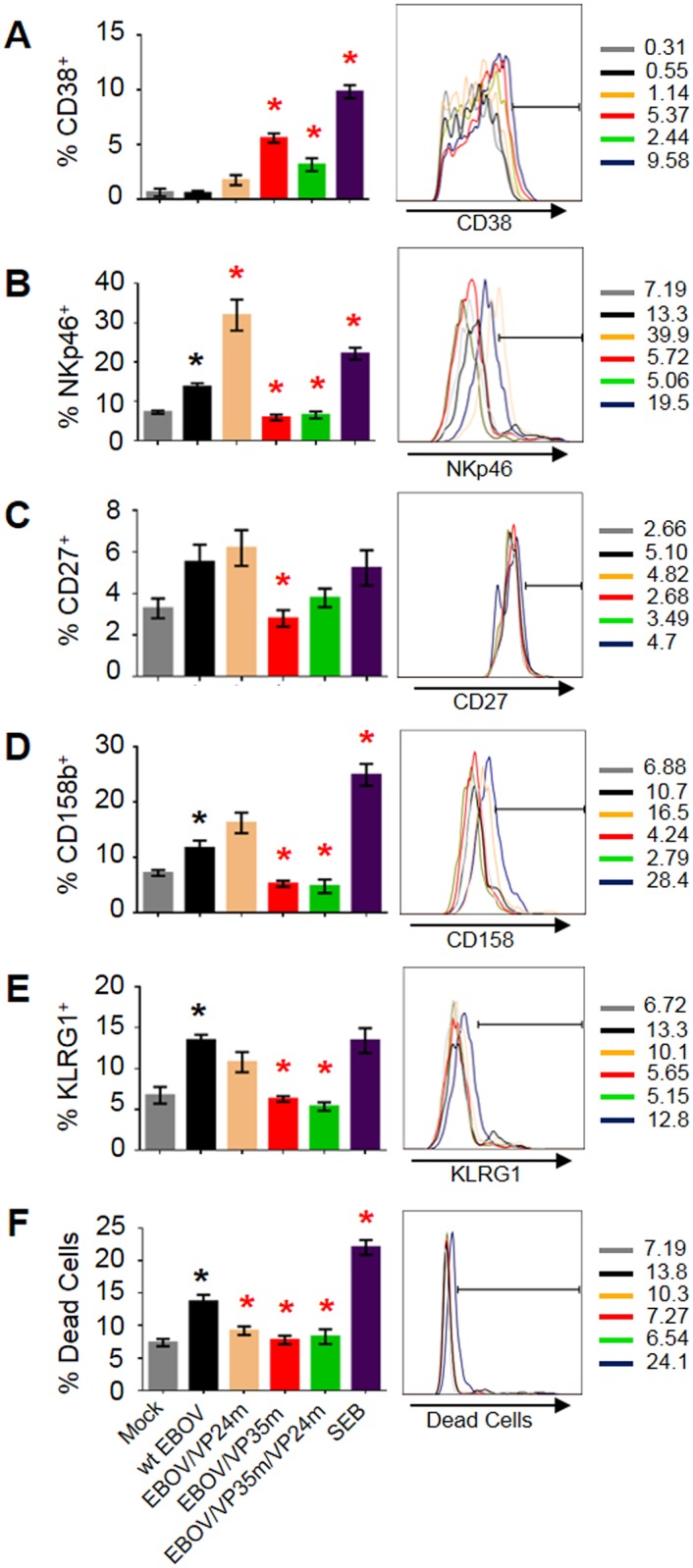
VP35 and VP24 IID suppress activation of NK cells. PBMCs were infected with wt EBOV or the mutated viruses, and gated CD56^+^CD3^-^ NK cells were analyzed for the indicated markers of activation, inhibition or dead cell markers: **A**, CD38; **B**, NKp46; **C**, CD27; **D**, CD158b; **E** KLRG1; **F**, Live/Dead. Mean values based on 3 donors with SE are shown. Statistically significant differences (P<0.05) for wt EBOV as compared to mock are indicated with black asterisks, and for the mutated viruses as compared to wt EBOV are indicated with red asterisks. Percentages of cell populations are indicated next to each histogram.

The primary role of NK cells is the continuous surveillance of “stressed” cells, which is regulated by detection of both activating and inhibitory signals, as well as by cytokines. One of the major molecules, whose expression affects activation of NK cells, is MHCI; reduced MHCI expression levels in virus-infected cells are sensed by NK receptors, which typically result in activation of NK cells [79, 80]. In addition, IFN-I is required for the optimal NK cell response and promotes the activation and effector functions of these cells [81]. As noted above, our previous study demonstrated that disabling of IID effectively unblocks maturation of EBOV-infected DCs, as evidenced by increased expression of multiples maturation markers, although expression of MHCI was not tested, and increased expression of IFN-I [50]. We therefore examined the effects of IID on NK function in PBMCs by analyzing expression of both activation and inhibitory markers on CD56^+^CD3^-^ NK cells. We tested expression of the two activation markers: CD38 (Fig 10A), which triggers cytolytic response [82] and NKp46 (Fig 10B), which is an immunoglobulin-like natural cytotoxicity receptor [83], and three inhibitory receptors: CD27 (Fig 10C), which is expressed by mature cytotoxic effector NK cells [84], CD158 killer immunoglobulin-like receptor (Fig 10D), which inhibits NK cytotoxicity [85] and the lectin-like receptor KLRG1 (Fig 10E) [86]. Paradoxically, infection with wt EBOV increased the percentages of NK cells positive for the activating marker NKp46 and all inhibitory markers tested, as well as the percentage of dead NK cells (Fig 10F). Disabling of VP24 IID resulted in a strong increase in the percentages of cells expressing NKp46, and a limited non-statistically significant increase of CD38, and reduced the percentages of dead cells. On the other hand, disruption of VP35 IID resulted in strongly (10.1-fold versus wt EBOV) increased expression of CD38 but not NKp46, which showed an opposite trend perhaps due to altered signaling events. Furthermore, it reduced expression of all three inhibitory receptors. These data suggest, based on multiple activating and inhibitory receptors, that both IIDs suppress activation of NK cells, with the exception of the effect of VP35 IID on the activating receptor NKp46.

## Discussion

Our findings provide new insights into functional role of IID in EBOV pathogenesis and identify their role as suppressors or modulators of both the innate and adaptive cell-mediated immune responses. We show that EBOV infection of PBMCs resulted in only a limited activation of T cells, while disabling of VP35 IID significantly increased their activation (Fig 2). As lymphocytes, along with NK cells, are refractory to EBOV infection, we hypothesized that the IID-associated effects are indirect and result from interaction of these cells with other cells susceptible to the virus, such as DCs. To identify the role of DCs, two co-culture systems were used, which included DCs infected with the panel of viruses and CMV-stimulated T cells (Fig 3A–3C), and its modified version with expanded CMV responder T-lymphocytes (Fig 3D and 3E). Analysis of supernatants of CMV-peptide stimulated DCs infected with wt EBOV co-cultured with expanded responder T-lymphocytes demonstrated that most of the cytokines and chemokines analyzed were below that observed in mock-infected control (Figs 2E and 4A). In addition, analysis of supernatants of wt EBOV-infected cultures where indicative of Th2 skewed response, as IL-5 and IL-13 were elevated. This is consistent with the induction of Th2 response in EBOV patients [87] and in macaques infected with Marburg virus [88], which similarly to EBOV belongs to the family *Filoviridae* and causes human infections with high case fatality rates. Supernatants of EBOV/VP24m-infected cells also demonstrated elevated levels of IL-13. However, flow cytometry analysis of T cell phenotypes of CD4^+^ T-cells from CMV-responder assays indicated that disabling of VP24 IID promotes expression of Th1-associated cytokines (Fig 3E) in addition to Th2, suggesting induction of a mixed Th1/Th2 response. Strikingly, flow cytometry analysis also demonstrated that disabling of VP35 IID results in the significant expansion of activated Th1 population suggesting that VP35 IID limits the capacity of EBOV-infected DCs to initiate the Th1 response. Consistent with that, analysis of EBOV/VP35 IID supernatants demonstrated several fold reduced levels of IL-5 and IL-13 indicating induction of a uniformly Th1-like response. Overall, the cytokine analysis provides a clear indication regarding the inhibitory effects of VP35 and VP24 IIDs, as their disruption by point mutations dramatically increased cytokine/chemokine secretion. A clear cumulative effect was observed in EBOV/VP35m/VP24m cultures as the levels of virtually all cytokines and chemokines were elevated in comparison to either single mutant. This finding suggests that activation of immune cells and their migration to sites of infection may be severely impaired *in vivo* due to the presence of IIDs. We note that in T cell activation analyses, that the effect of VP24 IID in the double mutant often countered the effects of the VP35 IID in a single mutant (Figs 2 and 3), which is consistent with our recent transcriptome analysis of DCs infected with the panel of viruses [51]. In general, the EBOV/VP24m mutant exhibited a phenotype consistent with more moderate effects in comparison with EBOV/VP35m. Interestingly, while the effects of VP35 IID on activation and proliferation of T cells in the DC-T cell co-cultivation system (Figs 2C, 2D, 3B, 3C and 7A) were strong, the effects of VP24 IID were minimal. We therefore expanded CMV-specific CD4^+^ T cells that resulted in not only better identification of the effects of VP35 IID, but also demonstrated the effects of VP24 IID (Figs 3E and 7B).

The limited formation of immunological synapses in co-culture experiments with wt infected DCs (Fig 6) demonstrated the mechanism by which VP35 effectively blocks the development of an adaptive immune response. As noted above, previous studies demonstrated that infection of DCs with wt EBOV results in their aberrant maturation. Hence, DC-associated ligands required for the formation of synapses are likely inadequately expressed resulting in fewer immunological synapse formations between DCs and T-cells. This reduction results in an inability to reach a signal transduction threshold necessary for cellular activation. This presumption is consistent with both the limited T cell activation observed in co-cultures experiments and the altered phosphorylation cascade profiles when T-cells were cultured with wt EBOV-infected DCs (Fig 8). The block in immunological synapses formation and the limited T cell activation were highly correlative with the presence of functional VP35 IID in wt EBOV, as disabling the VP35 IID reversed the suppressive effects.

The EBOV VP35 IID-mediated suppression of cytosolic sensing and induction of IFN-I response, which otherwise would induce an antiviral state are well established [reviewed in reference [47]. Thus both the lack of proper DC maturation and impairment in the development of an antiviral state blunt the initiation of a T cell response to EBOV infection. Interestingly, the suppression of IFN-I signaling and the prevention of IFN-I release *per se* during EBOV infection appeared to play only a limited role in the suppression of DCs maturation by the IID (Fig 5). On the other hand, transfer of conditioned media from DCs infected with EBOV/VP35m, but not wt EBOV, to DC-T cell co-cultures effectively stimulated secretion of IFNγ by T cells (Fig 4C and 4D), despite the block of IFN signaling by intact VP24 IID present in the virus. These data suggest that that some cytokines, such as TNFα whose expression was unblocked by disabling VP35 IID (Fig 4A) could contribute the observed DC maturation. More studies are required to mechanistically connect the IFN-inhibiting effects of EBOV IIDs with their effects on maturation of DCs and ultimately phosphorylation of TCR-associated adaptors and downstream signaling molecules.

These studies also demonstrate aberrant B cell and NK cell activation by EBOV, thus suggesting the global impairment of the adaptive and innate cell-mediated immune responses by IIDs. As indicated above, B-cell function and maturation are highly affected by IFN-I stimulation in the presence of cognitive antigen (35, 40–42). Previous findings have indicated that survivors of EBOV infection develop and maintain antigen specific adaptive immune responses, which included both CTL and humoral responses but did not include direct examination of antigen-specific B-cell function [89, 90]. We demonstrated an overall increase in the percentages of class switched and post class-switched memory B-cells in response to disruption of VP35 IID (Fig 9). These data suggest a suppressive effect of VP35 IID on class switching and identify the role of IFN-I in B-cell differentiation. Disruption of VP35 IID also led to an increase in the overall percentage of B-cells positive for markers associated with plasma cells. Furthermore, consistently with *in vivo* data demonstrating loss of NK cells in EBOV-infected macaques [4], infection of PBMCs with wt EBOV appeared to increase the rate of NK cell death. Similarly to our findings regarding increased functional activity, proliferation and differentiation of lymphocytes associated with disruption of VP35 IID, the mutation altered the expression of several NK cell markers in a direction generally corresponding to a greater cytotoxicity, and also reduced NK cell death (Fig 10). Intriguingly, disruption of VP24 IID dramatically increased the percentage of NK cells expressing the natural cytotoxicity receptor NKp46.

We note that this study was entirely performed with primary human immune cells from donors, which allowed us to detect the magnitude of the effects on cells with different genetic background, as can be seen by a relatively high donor-to-donor variability, and avoid producing skewed results related to a specific genetic background of inbred mice, such as the Th2-skewed response in BALB/c mice [91]. Despite that, the inhibition of activation of T cells co-cultured with EBOV-infected DCs contrasts the T cell activation in EBOV patients [13]. The most obvious difference is that most of CD8^+^ T cells in patients were positive for Ki-67, while only 12% of CD4^+^ T cells in our study were Ki67^+^ (Fig 7C). This discrepancy can be explained by a much greater increase in the numbers of Ki67^+^ CD8^+^ T cells as compared to Ki-67^+^ CD4^+^ T cells, as demonstrated with human immunodeficiency virus infection [92]. In addition, activation, including non-antigen specific activation, of T cells in infected patients by other types of cells not included in our studies, or by stimulation related to high doses of EBOV-specific antibodies administered to patients may contribute to the observed *in vivo* activation through an unknown mechanism.

These studies provide evidence of the dual role of the VP35 and VP24 IIDs in the pathogenesis of EBOV. While IIDs are intimately linked to the ability of EBOV to block IFN-I production and signaling, they also counter the ability of DCs to initiate the adaptive immune response. The lack of DC maturation following EBOV-infection presents a significant obstacle in development of the adaptive immune response but also may render immune cell populations susceptible to premature cell death due to aberrant signal transduction and/or the absence of survival signals. Importantly, the suppressive effect of IIDs is not limited to T and B cells, which are the central components of the adaptive response, but also extends to NK cells, the key players in cell-mediated innate immune response. Taken together, these findings suggest global suppressive effects of EBOV IIDs on cell mediated response, and also indicate the potential benefits of blocking the immunosuppressive effects of IIDs as a potential therapy for EBOV-infection.

## Materials and Methods

### Work in biosafety level 4 (BSL-4) containment

All work with EBOV was performed in BSL-4 facilities of the Galveston National Laboratory. Flow cytometry was performed either in BSL-4 using the Canto-II instrument (BD Biosciences), or cells were treated with 4% paraformaldehyde in PBS for 48 hours according the UTMB standard operating procedure and removed from BSL-4 for analysis with LSRII Fortessa flow cytometer (BD Biosciences) available at the UTMB Flow Cytometry Core Facility. Cells for confocal microscopy were placed on slides, stained, fixed in 4% paraformaldehyde for 24 hours, which was replaced with a fresh solution, incubated for additional 48 hours, and taken out of BSL-4. To remove supernatants of EBOV-infected cells from BSL-4, they were gamma-irradiated with the 5 Mrad dose according the UTMB standard operating procedure protocol. Staining and mounting procedures are described below. The staff had the U.S. government permissions and appropriate training for work with EBOV.

### Viruses

Generation of the recombinant EBOVs carrying the mutation R312A in the VP35 IID or K142A in the VP24 IID, or both, each expressing GFP from an added gene, and the control GFP-expressing EBOV with no mutations was described previously [50, 51]. The viruses were propagated on Vero-E6 monolayers and quantitated by plaque titration as previously described [93].

### Study population

Peripheral blood nuclear cells (PBMC) were obtained from buffy coats from anonymous healthy adult blood donors from the UTMB blood bank according to a clinical protocol approved by the UTMB Institutional Review Board. Study population included both CMV-positive and CMV-negative individuals as tested using the Beckman Coulter PK CMV-PA System for qualitative detection of IgG and IgM antibodies to CMV.

### Stimulation with HCMV peptides

A pool of 138 CMV peptides (15-mers overlapping by 11 amino acid residues) was obtained from the NIH AIDS Research and Reference Reagent Program. Peptides were reconstituted at 1 mg/ml in DMSO and stored at -70°C in 200 μl aliquots and used to stimulate PBMC or dendritic cells at final concentration of 2 μg/ml.

### Infection of PBMCs

Total PBMC from CMV^+^ donors were resuspended at 1x10^6^ per ml using 50 ml conical tubes and inoculated at an MOI of 2 PFU/cell with the recombinant strains of EBOV depicted in Fig 2A and simultaneously stimulated with 2 μg/ml of CMV pp65 peptides in media containing 10% human serum (Corning, Celgro) at 37°C, 5% CO_2_. Staphylococcal Enterotoxin B (SEB) (Sigma Aldrich) was used as a positive control at a final concentration of 2 μg/ml. Additional controls included mock-infected cells and cells stimulated with 15-mer CMV pp65 peptides only. After 4 hours of incubation, cells were washed twice by centrifugation at 200 x g for 5 min with 2% human serum media and cultured at 1x10^6^ per ml in Advanced RPMI 1640 medium (Gibco, Life technologies) supplemented with 10% human serum (Gemini Bio-Products), 2 mM L-glutamine, 200 IU/ml penicillin, and 200 μg/ml streptomycin sulfate (Invitrogen) in 6-well plates.

### Isolation of monocytes and generation of monocyte-derived immature dendritic cells (DC)

PBMC were isolated by density gradient centrifugation (histopaque; Sigma life science). CD14^+^ monocytes were purified by positive selection using anti-CD14 monoclonal antibody-coated magnetic microbeads according to the manufacturer’s instructions (Quadro Macs; Miltenyi Biotech). CD14^+^ monocytes were cultured in T-225 flasks (Corning Incorporated, Costar) at 6 x 10^5^ cells per ml in Advanced RPMI 1640 medium (Gibco, Life Technologies) supplemented with 10% heat-inactivated bovine serum (Quality Biologicals), 2 mM l-glutamine (Invitrogen), 0.05 mM β-mercaptoethanol, 50 ng/ml granulocyte-macrophage colony-stimulating factor (R&D Systems), 16 ng/ml interleukin-4 (R&D Systems), 200 IU/ml penicillin, and 200 μg/ml streptomycin sulfate (Invitrogen). The cells were incubated for 7 days at 37°C, 5% CO_2_ as described previously [50].

### Infection of DCs

Immature DCs generated as described above were harvested, infected with the panel of EBOVs and stimulated with CMV pp65 peptides either simultaneously or 24 hours following infection. After 4 hours of peptide stimulation, cells were washed twice and co-cultured with CFSE (Molecular Probes, Life Technologies)-labeled autologous PBMC or purified CD4^+^ T cells at 37°C, 5% CO_2_ for 7 days. The DC: lymphocyte ratio was 1:10 (2 x 10^5^ DC: 2 x 10^6^ lymphocytes) in 2 ml of Advanced RPMI 1640 medium. For assays involving purified CD4 or CD8 T cells, these cell populations were purified by negative selection using a primary cocktail of antibodies conjugated to biotin and secondary anti-biotin antibody conjugated to magnetic microbeabs in order to deplete non-CD4^+^ or non-CD8^+^ T cells including γ/δ T cells, B cells, NK cells, DC, monocytes, granulocytes and erythroid cells.

### Lentiviral-mediated transduction of DCs

Lentiviral vectors encoding wt and R312A mutant VP35 were prepared as previously described [94]. Briefly, plasmids encoding the lentivirus, HIV-Gag-pol, VSV-G and SIV-Vpx were transfected into 293T using PEI (Sigma Aldrich) transfection reagent. Cell monolayers were incubated overnight, fresh medium was added, and monolayers were incubated for additional 48 hours. Thereafter cell-free supernatants were collected and lentiviral vectors were titrated in 293T cell monolayers. DCs were transduced twice with ~8 hour culture period between the addition of lentiviral stocks. Following three days of culture, mock or medium containing CMV peptides were added. Autologous CD4^+^ T-cells were added to transduced T-cells at a 1:1 ratio, and the percentages of IFNγ^+^ CD4^+^ T-cells were determined 24 hours after initiation of co-culture.

### Stimulation and staining

After 7 days of culture, cells were harvested, washed and 2 x 10^6^ cells were stimulated for 6 hours in culture medium with 10 μg/ml Brefeldin A (Sigma-Aldrich), 0.7 μg/ml monensin (GolgiStop, BD Biosciences), 1 μg/ml anti-CD28 (BD Biosciences), 1 μg/ml anti-CD49d (BD Biosciences), 20 μg/ml DNase (Calbiochem) and 2 μg/ml of 15-mer CMV pp65 peptides. PMA (Sigma Aldrich) at 20 μg/ml and ionomycin (EMD Chemicals) at 1 μg/ml were also used as an additional positive control. Following stimulation, cells were washed 2 x with wash buffer (PBS, 1% FBS, 0.02% sodium azide) followed by PBS. CD4^+^ T cells were stained extracellularly with anti-CD3 antibodies labeled with anti-CD3 BD Horizon Brilliant Ultraviolet 395 (clone UCHT1, BD Biosciences) and anti-CD4 PE-CF594 (clone RPA-T4, BD Biosciences); for analysis of activation, cells were also stained with CD69-PE/Dazzle (clone FN50, BioLegend) or anti-Ki67-brilliant violet 421 (Clone B56, BD Biosciences). CD8^+^ T cells were stained extracellularly with anti-CD3 BD Horizon Brilliant Ultraviolet 395 (clone UCHT1, BD Biosciences) and anti-CD8 BD Horizon PE-CF594 (clone RPA-T, BD Biosciences). Both CD4^+^ and CD8^+^ T cell subsets were also stained extracellularly with Live/Dead Fixable Aqua or Near-Infra Red (Invitrogen) to discriminate dead cells by flow cytometry for 30 minutes at 4°C. Following extracellular staining, cells were washed, fixed and permeabilized with CytofixCytoperm (BD Biosciences) according to manufacturer’s instructions. CD4^+^ and CD8^+^ T cells were stained intracellularly with the following antibodies: anti-IFNγ-PE (clone B27), anti-IL2-allophycocyanin (APC) (clone MQ1-17H12), anti-IL-4-peridinin chlorophyll protein PerCP)/Cy5.5 (clone 8D4-8), anti-IL17a-BD Horizon V450 (clone N49-653) and -TNFα-Alexa Fluor 700 (clone Mab11); all from BD Biosciences. Flow cytometry analysis of DCs for HLA-DR was performed with anti-HLA-DR-PE/Dazzle 594 (clone L243, BioLegend). B-cell subset staining was performed as follows: anti-CD19-PerCP/Cy5.5 (clone HIB19, BioLegend), anti-IgD-PE/CF594 (clone IA6-2, BD Biosciences), anti-IgM-brilliant violet 786 (clone R6-60.2, BD Biosciences), anti-CD27-brilliant violet 510 (clone M-T271, BioLegend), anti-CD24-PE (clone ML5, BioLegend), anti-CD20-Alexa Fluor 700 (clone 2H7, BioLegend), anti-CD38-APC (clone HIT2, BioLegend), anti-IgG-Brilliant Violet 421 (clone G18-145, BD Bioscience), anti-CD138-fluorescein isothiocyanate (FITC) (clone DL-101, BioLegend). To analyze CD56^+^CD3^-^ NK cell function/activation, the following antibodies were used: anti-CD38-Alexa Fluor 488 (clone HIT2, BioLegend), anti-NKp46-Brilliant Violet 786 (clone 9E2, BD Bioscience), anti-CD27-Brilliant Violet 510 (clone M-T271, BioLegend), anti-CD158B-APC (clone DX27, BioLegend), anti-KLRG1-PE-CF594 (clone 14C2A07, BioLegend), anti-CD56-Brilliant Violet 421 (clone HCD56, BioLegend) and anti-CD3-Brilliant Ultraviolet 395 (clone UCHT1, BD Biosciences). Cells were subsequently washed twice with Perm/Wash and one time with PBS, fixed with 4% paraformaldehyde (Polysciences) and taken out of BSL-4 according to an approved protocol. Cells were washed, resuspended in PBS and 200,000 to 500,000 events were acquired on the BD LSR II flow cytometer (BD Biosciences). For data analysis, FlowJo version 10 (Tree Star) and SPICE (National Institute of Allergy and Infectious Diseases) software was used to create Boolean gate arrays that allowed us to determine the frequency of 32 possible response patterns based on the five cytokines tested.

### Proliferation

PBMCs from CMV-positive donors were resuspended in RPMI media supplemented with 2% human AB serum (Corning, Celgro), and labeled with 5 μM CFSE (Molecular Probes, Life Technologies). CFSE-labeled PBMCs were inoculated with the recombinant strains of EBOV at an MOI of 2 PFU/cell, with or without simultaneous stimulation with 2 μg/ml of CMV pp65 peptides, SEB treated, or mock treated for 4 hours. PBMCs were washed twice to remove virus inoculum and cultured at a concentration of 2 x 10^6^ cells/ml for 7 days in Advanced RPMI medium supplemented with 10% human AB serum, 2 mM L-glutamine, 200 IU/ml penicillin, and 200 μg/ml streptomycin sulfate (Invitrogen) in 6 well plates. PBMCs were harvested, stained extracellularly with the following antibodies: anti-CD3 PE-Cy7 (clone SK7, BD Biosciences), anti-CD4-PerCP/Cy5.5 (clone SK3, BD Biosciences), anti-CD8 APC-Cy7 (clone SK1, BD Biosciences), and Live/Dead-Far Red Dead cell stain (Molecular Probes, Life Technologies). Cells were subsequently washed, fixed with 4% paraformaldehyde and removed from the BSL-4 according to an approved protocol. Cells were washed with PBS and analyzed by flow cytometry on BD LSR II.

### Isolation and expansion of CMV-specific T-lymphocyte responders

PMBCs were pulsed with CMV peptides as previously described. Following 24–48 hours, CD137^+^ T-lymphocytes were isolated using magnetic bead separation in accordance with the manufacturer’s protocol (Miltenyi). Cells were expanded for 8 days in complete RPMI (Gibco)/10% Human serum (Cellgro) media supplemented with recombinant IL-2 (10 ng/ml) and IL-7 (10 ng/ml) (R&D Systems) and Dynabeads Human T-Activator CD3/CD28/CD137 beads (ThermoFisher Scientific) to promote the expansion of CMV-specific responders. DCs were infected at an MOI of 3 and pulsed overnight with CMV peptides. Expanded responders were cultured with 2x10^5^ autologous DCs at a 1:1 ratio for an additional 24 hours. Intracellular staining was performed as described previously.

### Western blotting

Frozen CD4^+^ T-cells were recovered at 37°C for 2 hours prior to co-culture with autologous DCs infected overnight with wt or mutant EBOVs (MOI of 3) at a 1:1 ratio. CD4^+^ T-cells were harvested after 4 days of co-culture, and analyzed by Western Blotting. Briefly, cell pellets were lysed in Laemmli lysis buffer (Invitrogen), separated on 4–12% SDS-PAGE gradient gels (ThermoFisher Scientific) and transferred to nitrocellulose membranes using the I-blot system (ThermoFisher Scientific). Thereafter blots were incubated with primary antibodies provided in the T-cell Signaling Antibody Sampler and the Pro-Survival Bcl-2 Family Antibody Sampler Kits (Cell Signaling Technologies). GAPDH was used as an internal loading control (Cell Signaling Technologies). HRP-conjugated secondary antibodies (Santa Cruz Biotech) were used for chemiluminescent detection on Hyperfilm (Amersham).

### Cytokine analysis

For analysis of cytokines and chemokines in CMV-responder—DC co-culture assays, cell-free supernatants were irradiated at 5 Mrad, stored at -80°C and shipped to Eve Technologies on dry ice. Undiluted samples were analyzed using Human Cytokine/Chemokine Array 65-Plex Panel. Heatmaps of values normalized to mock-infected samples were generated using GENE-E software (Broad Institute). In some experiments, conditioned media were collected from DC-T-cell co-culture plates, clarified of cellular debris by low-speed centrifugation and stored at -80°C. Following overnight recovery, CD4^+^ T-cells were plated in 96-well plates in 100 μl of RPMI1640 medium supplemented by 10% FBS, followed by the addition of 150 μl of conditioned media. Cells were incubated overnight followed by the addition of brefeldin A and monensin for an additional 5 hours. Cells were then stained as previously described.

### IFNAR2 blockade and exogenous IFNα/IFNβ

In experiments requiring IFNAR2 blockade, DC were pre-incubated with 30 μg/ml of blocking antibody specific to IFNAR subunit 2 (CD118; PBL Assay Science) or mouse immunoglobulin G2a (IgG2a; R&D Systems) as an isotype control (the endotoxin levels in the two antibody preparations were <1 and 0.1 endotoxin units per 1 μg of antibody, respectively), for 1 hour before virus inoculation. In the experiments involving exogenous IFNs, recombinant human IFNα2a or IFNβ1a (PBL Assay Science) was added to DCs at 24 hours after infection at a final concentration of 1,000 IU/ml or 800 IU/ml, respectively, and after additional 20 hours, cells were analyzed by flow cytometry. DC were analyzed for cell surface expression of markers of maturation by flow cytometry at 40 hours post-infection. Most DCs were collected by pipetting, and the remaining cells, which were attached to the bottom of the plates, were collected by applying staining buffer (PBS containing 2% fetal bovine serum and 2 μM EDTA). Cells were pelleted by centrifugation at 200×*g* at 4°C for 5 minutes, buffer was removed, and the pellet was re-suspended in staining buffer. DCs were incubated for 20 min on ice in the dark with the monoclonal antibodies anti-CD86-PerCP-Cy5.5 (clone FUN-1), anti-CD80-APC-H7 (clone L307.4), and anti-CD54-PE (clone HA58). In addition, IgG2a-PerCp-Cy5.5, IgG1-APC-H7, and IgG1-PE were used as the respective isotype control antibodies (all from BD Biosciences). Following incubation, cells were washed three times with the staining buffer and re-suspended in 200 μl of the same buffer. The Far Red fluorescent dye (Invitrogen) was used to evaluate cell viability by flow cytometry. Data were acquired using a flow cytometer FACSCanto II in BSL-4 facility or fixed with formalin as described above, taken out of BSL-4 and analyzed with BD LSR flow cytometer. Data were analyzed using FlowJo 7.6.1 software (Tree Star).

### Confocal microscopy

Suspensions of DCs and lymphocytes were fixed with 4% paraformaldehyde for 15 minutes and washed 3 times with PBS. Cells were then pelleted by centrifugation at 600xg for 6 minutes and resuspended in 50 μL of PBS. Cells were loaded on charged slides and dried overnight at 4°C. For staining, cells were rehydrated with PBS and permeabilized with 0.5% Triton X100 solution (ThermoFisher Scientific) in PBS for 15 minutes, washed with PBS, incubated with 0.5 M glycine in PBS for 10 minutes at room temperature, and washed 3 times with PBS. Antigen blocking was performed using 5% donkey serum (Sigma Aldrich) diluted in stain buffer (1% BSA and 0.1% Triton X100 in PBS) for 30 minutes. Mouse monoclonal antibody targeting HLA-DR (ThermoFisher Scientific, clone 7.3.19.1, 1:10 dilution) and goat polyclonal targeting CD3ε (Santa Cruz, 1:50 dilution) were used as primary antibodies and diluted in stain buffer as indicated. After 1 hour incubation at 37°C, slides were washed 3 times in washing buffer (0.1% Triton X100 in PBS), incubated with a mixture of two secondary antibodies: donkey anti-mouse conjugated with Alexa Fluor 647 (ThermoFisher Scientific) and donkey anti-goat conjugated with Alexa Fluor 594 (ThermoFisher Scientific) both diluted at 1:200 in stain buffer for 1 hour. Next, washed cells were incubated with 6-diamin-2-phenylindole dihydrochloride (DAPI) (ThermoFisher Scientific) at 1 μg/ml for 2 minutes, and washed 3 times in PBS. Slides were then fixed in 4% paraformaldehyde and removed from BSL-4. Slides were washed in 0.5 M glycine, washed in PBS, and mounted with coverslips using PermaFluor mounting medium (ThermoFisher Scientific). Infected cells were identified by expression of GFP encoded by the recombinant viruses. Slides were analyzed by laser scanning confocal microscopy using Olympus FV1000 confocal microscope. Lasers with 405 nm wavelengths were used for DAPI excitation, 488 nm for GFP, 543 for Alexa Fluor 594, and 635 nm for Alexa Fluor 647. This was followed by counting of immunological synapses, defined as colocalizations of CD3 and MHC-II (HLA-DR). For each experimental group, the data presented are based on count of at least 10 confocal imaging acquisitions. Results of the count were expressed as numbers of synapses per 100 cells.

### Statistics and graphs

Statistical analyses and generations of graphs were performed using GraphPad Prism version 6.05 (GraphPad Software). Statistical significances were calculated using a paired T-test. Statistical significance was set at *p* < 0.05.

## Supporting Information

S1 Fig The VP35 IID reduces Th1 response. CFSE-labeled total PBMC were inoculated with the indicated viruses in presence of CMV peptides and cultured for 7 days. Cells were re-stimulated with peptides, intracellularly stained for the indicated cytokines, and analyzed by multicolor flow cytometry. Representative primary data showing CD4^+^ T cells gated on total (top of each panel) and CFSE^-^ (bottom) populations positive for IFNγ (**A**), IL-2 (**B**), TNFα (**C**), and populations positive for IFNγ and TNFα (**D**). Percentages of cytokine secreting cells are indicated in the upper right corner.
(TIF)

S2 Fig The VP35 IID reduces Th1 response: polyfunctional CD4^+^ T cell responses. Percentages of CD4^+^ T cells secreting a combination of multiple individual cytokines following infection of total PBMCs from 3 individual donors with the panel of viruses and CMV stimulation. Each bar indicates the percentage of CD4^+^ T cells expressing an indicated combination of the markers of activation (IFNγ, IL-2, IL-4, IL-17 and TNFα) as determined by the Boolean gating. **A.** Total CD4^+^ T cells. **B.** CFSE^-^ proliferating CD4^+^ T cells.
(TIF)

S3 Fig Effects of disabling IIDs on viral infectivity in DCs. **A**. Percentages of GFP^+^ CD1c^+^CD123^+^ DCs in PBMCs (**A**) or purified DCs (**B**) infected with the panel of viruses. Mean values of triplicate samples with SE from one of two independent experiments performed with different donors, which resulted in essentially same results. Statistically significant differences (P<0.05) for wt EBOV as compared to mock are indicated with black asterisks, and for the mutated viruses as compared to wt EBOV with red asterisks.
(TIF)

S4 Fig The VP35 IID-mediated reduction of Th1 response results from the deficient maturation of DCs. **A.** Representative primary flow cytometry data showing expression of the indicated cytokines by CD4^+^ T cells cultured with autologous DCs infected with the indicated viruses and simultaneously pulsed with CMV peptides gated on total CD4^+^ (top) and CFSE^-^ CD4^+^ (bottom) T cell populations. Percentages of cells positive for the indicated cytokines are indicated in the gate. **B.** Representative primary flow cytometry data showing secretion of the indicated cytokines by expanded CMV-specific T-lymphocyte responders, which were cultured with CMV-pulsed DCs infected with the indicated viruses.
(TIF)

S5 Fig Comparison of CMV peptide stimulation of DCs during infection versus 24 hours after infection. **A**. Percentages of GFP^+^ DCs. **B.** Percentages of IFNγ^+^ CD4^+^ T cells co-cultured with infected DCs. Mean values of triplicate samples with SE from one of two independent experiments performed with different donors, which resulted in essentially same results. No statistically significant difference was observed between DCs pulsed with CMV-peptides simultaneously or 24 hours following infection with the panel of viruses.
(TIF)

S6 Fig The VP35 IID-mediated reduction of Th1 response results from the deficient maturation of DCs: analysis of multiple effector functions of T cells. Percentages of CD4^+^ T cells secreting a combination of multiple individual cytokines following cocultivation of DCs pre-infected with the panel of EBOVs and stimulated with CMV peptides with autologous CD4^+^ T cells. Each bar indicates the percentage of CD4^+^ T cells expressing an indicated combination of the markers of activation (IFNγ, IL-2, IL-4, IL-17 and TNFα) as determined by the Boolean gating. **A.** Total CD4^+^ T cells. **B.** CFSE^-^ proliferating CD4^+^ T cells.
(TIF)

S7 Fig The VP35 IID-mediated reduction of Th1 response results from the deficient maturation of DC: primary data on multiple effector functions of T cells. Representative primary flow cytometry data showing expression of IFNγ and TNFα by CD4^+^ T cells co-cultured with DCs pre-infected with the indicated viruses and simultaneously pulsed with CMV peptides gated on total CD4^+^ (top) and CFSE^-^ CD4^+^ (bottom) T cell populations. Percentages of cells positive for the indicated cytokines are indicated in the gate.
(TIF)

S8 Fig Analysis of the role of VP35 IID in stimulation of T cells by delivery of wt or mutated VP35 by lentiviral vectors. DCs were transduced with lentiviral vector expressing wt or R312A mutant VP35, incubated with or without CMV peptides, cultured with CD4^+^ T cells, and the percentages of IFNγ^+^ cells were determined by flow cytometry. Results are normalized to the mean of mock samples. Mean values with SE based on triplicate samples from one of two independent experiments performed with different donors, which resulted in essentially same results. Statistically significant differences (p<0.05) for the mutated VP35 as compared to wt VP35 are shown with asterisks.
(TIF)

S9 Fig Role of IFN-I signaling and the released IFN in the suppressive effects of IIDs on DC maturation: expression of CD80 and CD54. **A.** Effect of IFNAR2 blockade. **B.** Effect of exogenously added IFNα and IFNβ. MFI for CD80^+^ or CD54^+^ mock-treated DC (black), or DC treated with IFNAR2 antibodies, IFNα, or IFNβ (red, blue, brown, respectively) are indicated in upper left corners. The experiment was performed two times with essentially similar results.
(TIF)

S10 Fig Effects of the VP35 IID on T-cell proliferation. DCs were infected with wt EBOV or the mutated viruses indicated at the top and cultured with autologous CFSE-labeled CD4^+^ or CD8^+^ T cells. Proliferated CD4^+^ (top) and CD8^+^ T cells (bottom) were analyzed by CFSE dilution assay; percentages of CFSE low or negative proliferating cells are indicated.
(TIF)

S11 Fig The VP35 and VP24 IID inhibit B-cell function and differentiation. **A.** Percentages of naïve B cells in PBMCs infected with wt or mutated EBOVs. A statistically significant difference (P<0.05) for wt EBOV as compared to mock is indicated with the black asterisk, and for SEB as compared to wt EBOV with the red asterisk. **B.** Representative primary data showing gating used to analyze memory B-cell subsets shown in Fig 10B and 10C. Percentage of each gated population is shown. **C.** Additional subgating flow plot used to identify plasmablasts in Fig 10D. Percentages of cell populations are shown for each quadrant; percentage values used for graphs in Fig 10D in the upper right quadrant are bolded.
(TIF)

S1 Table Percentages of total and proliferating (CFSE^-^) CD4^+^ T cells secreting total IFNγ, IL-2 or TNFα: wt EBOV values from Fig 2C. (DOC)

S2 Table Percentages of total and proliferating (CFSE^-^) CD4^+^ T cells secreting single IFNγ^+^ or a combination of multiple cytokines: wt EBOV values from Fig 2D. (DOCX)

S3 Table Percentages of total and proliferating (CFSE^-^) CD4^+^ T cells secreting total IFNγ, IL-2 or TNFα: wt EBOV values from Fig 3B. (DOCX)

S4 Table Percentages of total and proliferating (CFSE^-^) CD4^+^ T cells secreting single IFNγ^+^ or a combination of multiple cytokines: wt EBOV values from Fig 3C. (DOCX)

S5 Table Concentrations of cytokines and chemokines in supernatants of T cells co-cultured with DCs infected with the panel of viruses. (DOCX)

S6 Table Percentages of CD4^+^ and CD8^+^ T cell proliferation by CFSE dilution assay: wt EBOV values from Fig 7A. (DOCX)
