# Supplementary figures and images for: The Ebola Interferon Inhibiting Domains Attenuate and Dysregulate Cell-Mediated Immune Responses
Source: PLoS Pathog. 2016 Dec 8;12(12):e1006031. doi: 10.1371/journal.ppat.1006031 (PMC5145241; doi:10.1371/journal.ppat.1006031)

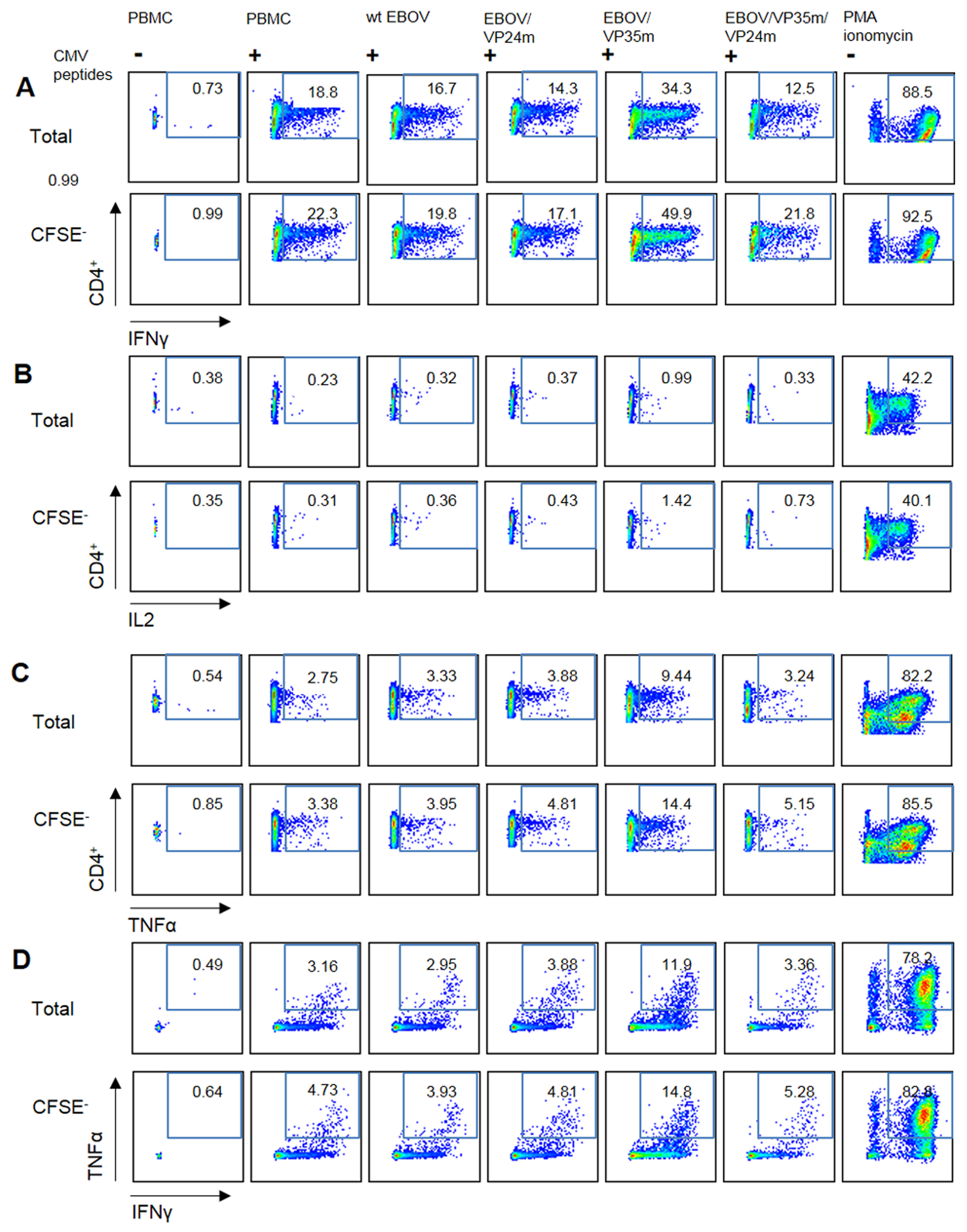

Supplement: S1 Fig — CFSE-labeled total PBMC were inoculated with the indicated viruses in presence of CMV peptides and cultured for 7 days. Cells were re-stimulated with peptides, intracellularly stained for the indicated cytokines, and analyzed by multicolor flow cytometry. Representative primary data showing CD4+ T cells gated on total (top of each panel) and CFSE- (bottom) populations positive for IFNγ (A), IL-2 (B), TNFα (C), and populations positive for IFNγ and TNFα (D). Percentages of cytokine secreting cells are indicated in the upper right corner. (TIF) [file ppat.1006031.s001.tif]

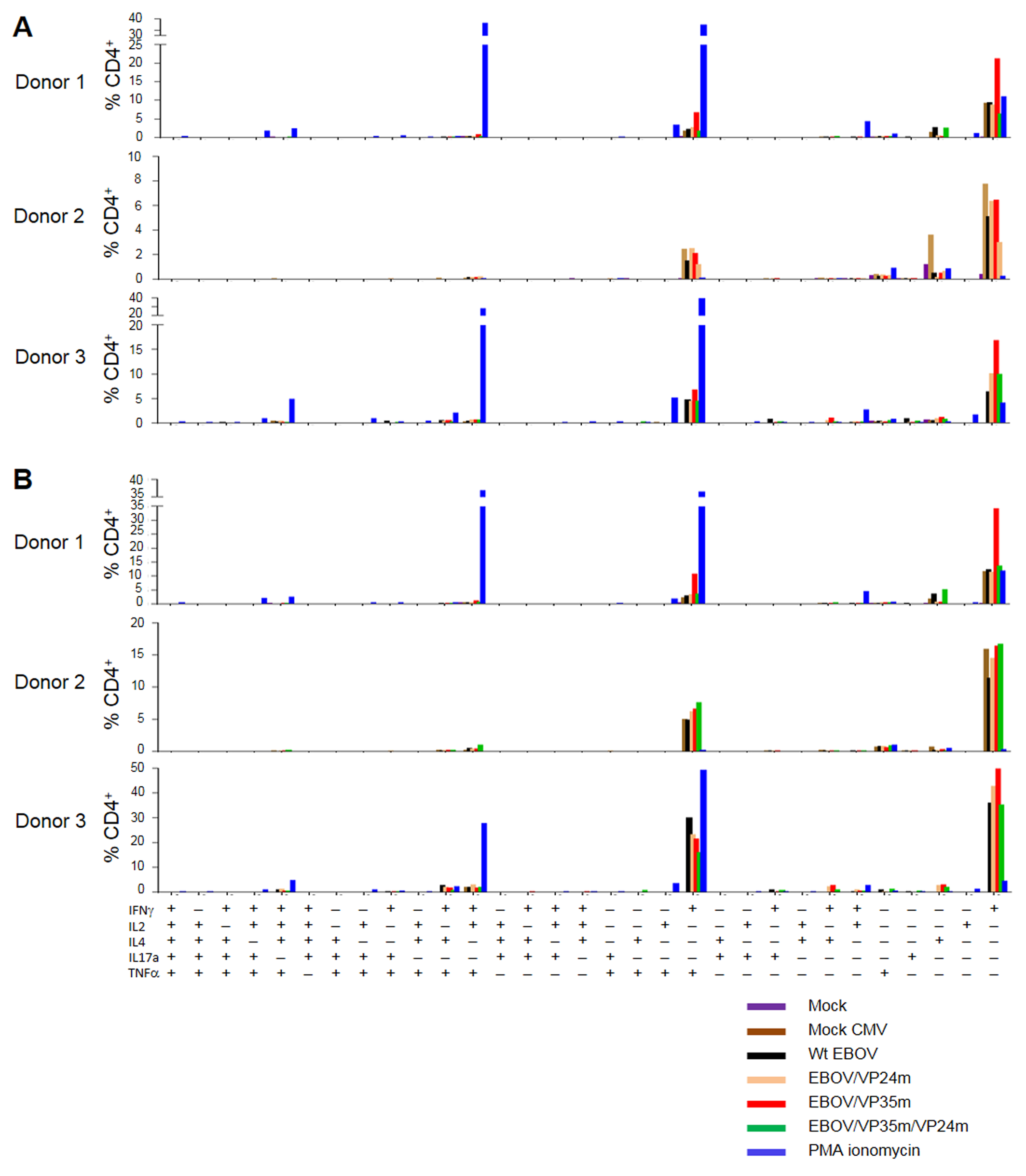

Supplement: S2 Fig — Percentages of CD4+ T cells secreting a combination of multiple individual cytokines following infection of total PBMCs from 3 individual donors with the panel of viruses and CMV stimulation. Each bar indicates the percentage of CD4+ T cells expressing an indicated combination of the markers of activation (IFNγ, IL-2, IL-4, IL-17 and TNFα) as determined by the Boolean gating. A. Total CD4+ T cells. B. CFSE- proliferating CD4+ T cells. (TIF) [file ppat.1006031.s002.tif]

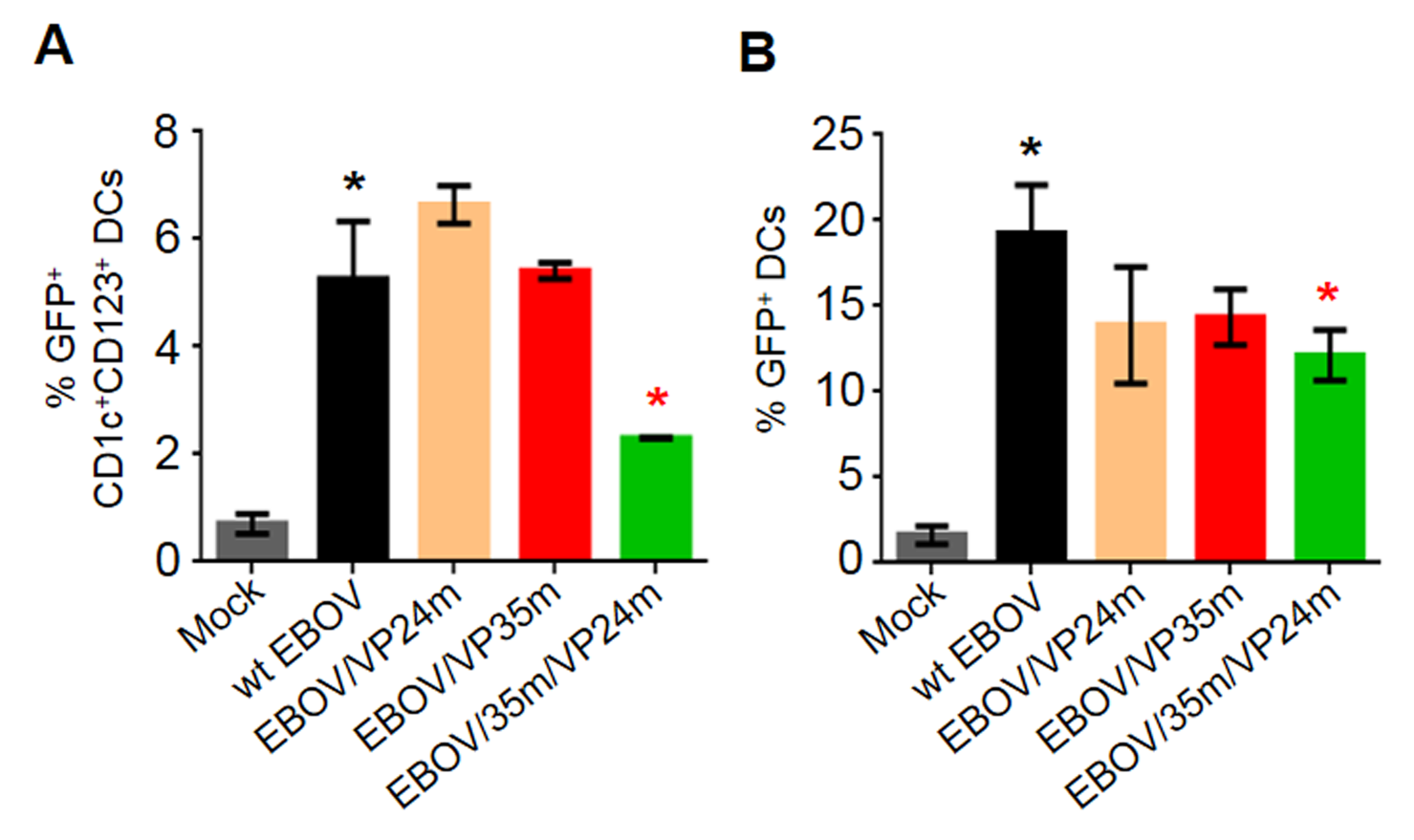

Supplement: S3 Fig — A. Percentages of GFP+ CD1c+CD123+ DCs in PBMCs (A) or purified DCs (B) infected with the panel of viruses. Mean values of triplicate samples with SE from one of two independent experiments performed with different donors, which resulted in essentially same results. Statistically significant differences (P<0.05) for wt EBOV as compared to mock are indicated with black asterisks, and for the mutated viruses as compared to wt EBOV with red asterisks. (TIF) [file ppat.1006031.s003.tif]

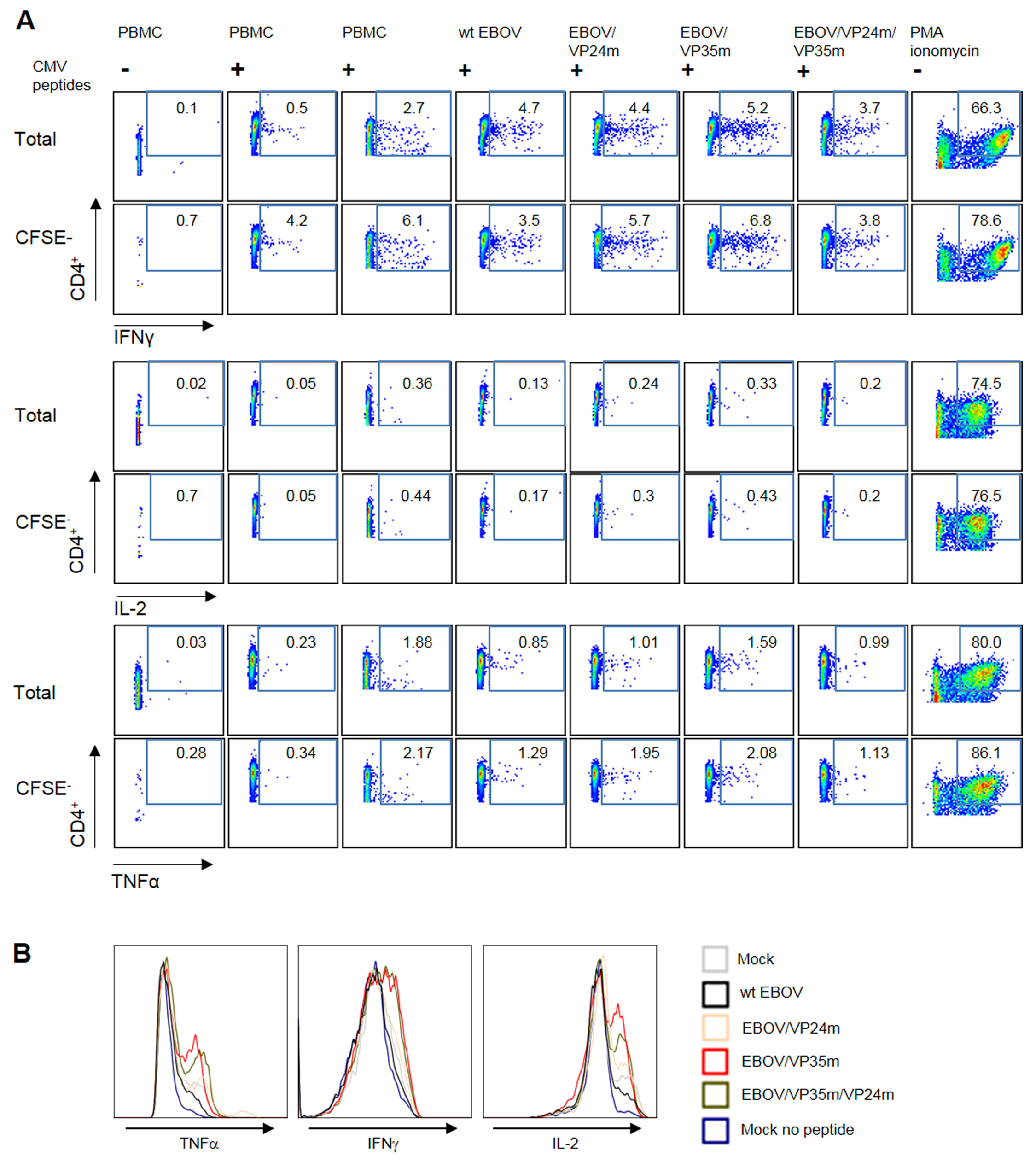

Supplement: S4 Fig — A. Representative primary flow cytometry data showing expression of the indicated cytokines by CD4+ T cells cultured with autologous DCs infected with the indicated viruses and simultaneously pulsed with CMV peptides gated on total CD4+ (top) and CFSE- CD4+ (bottom) T cell populations. Percentages of cells positive for the indicated cytokines are indicated in the gate. B. Representative primary flow cytometry data showing secretion of the indicated cytokines by expanded CMV-specific T-lymphocyte responders, which were cultured with CMV-pulsed DCs infected with the indicated viruses. (TIF) [file ppat.1006031.s004.tif]

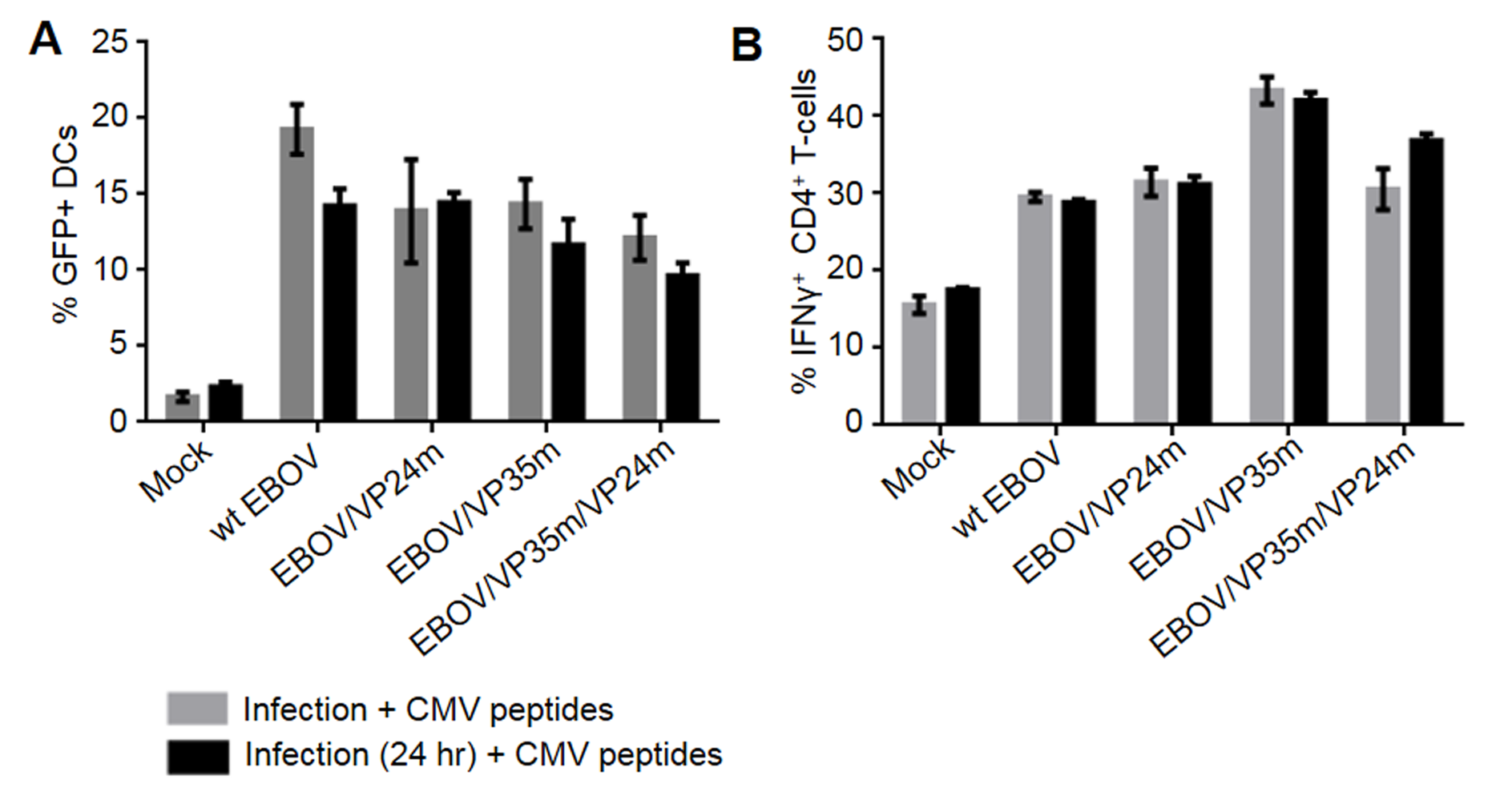

Supplement: S5 Fig — A. Percentages of GFP+ DCs. B. Percentages of IFNγ+ CD4+ T cells co-cultured with infected DCs. Mean values of triplicate samples with SE from one of two independent experiments performed with different donors, which resulted in essentially same results. No statistically significant difference was observed between DCs pulsed with CMV-peptides simultaneously or 24 hours following infection with the panel of viruses. (TIF) [file ppat.1006031.s005.tif]

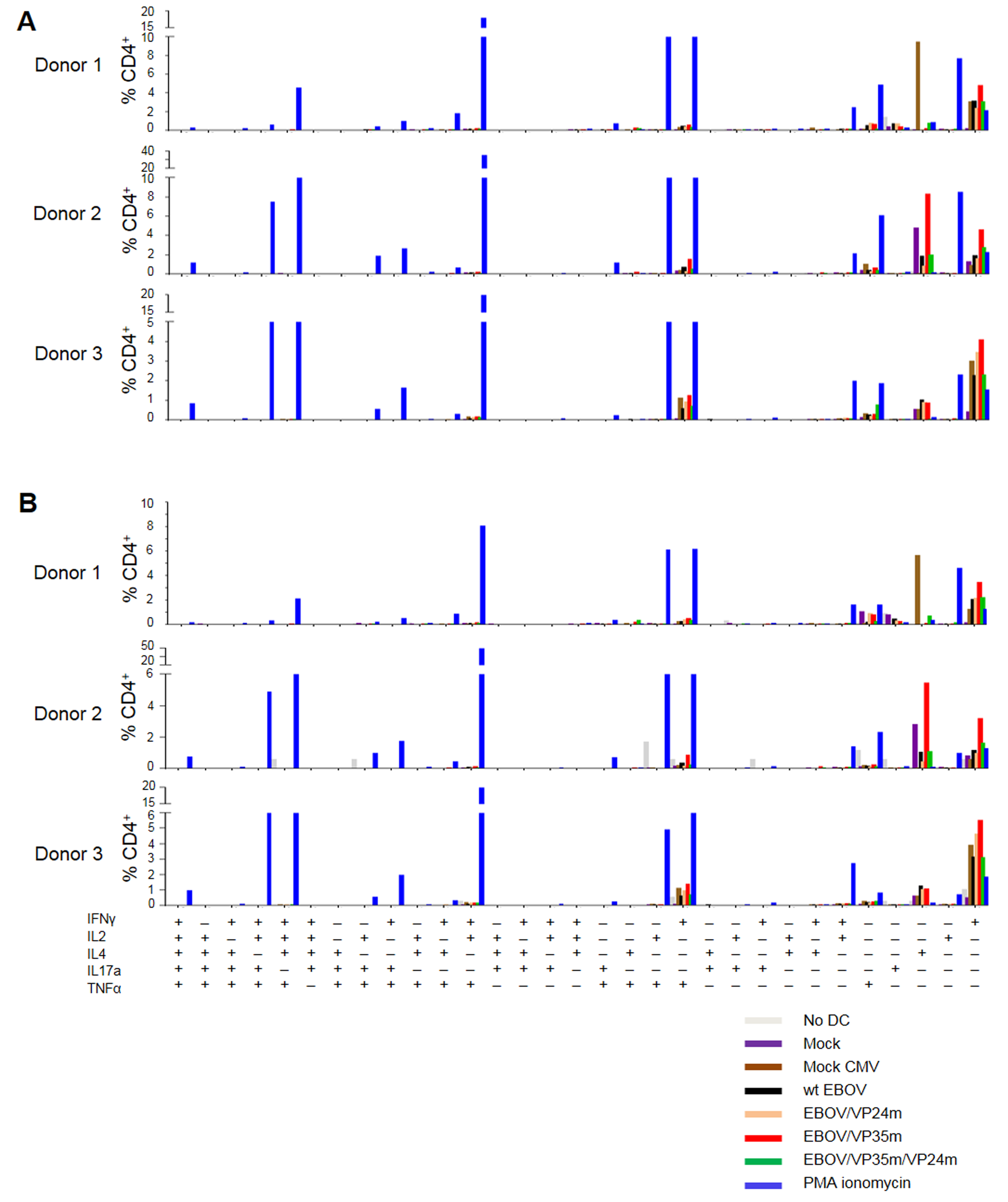

Supplement: S6 Fig — Percentages of CD4+ T cells secreting a combination of multiple individual cytokines following cocultivation of DCs pre-infected with the panel of EBOVs and stimulated with CMV peptides with autologous CD4+ T cells. Each bar indicates the percentage of CD4+ T cells expressing an indicated combination of the markers of activation (IFNγ, IL-2, IL-4, IL-17 and TNFα) as determined by the Boolean gating. A. Total CD4+ T cells. B. CFSE- proliferating CD4+ T cells. (TIF) [file ppat.1006031.s006.tif]

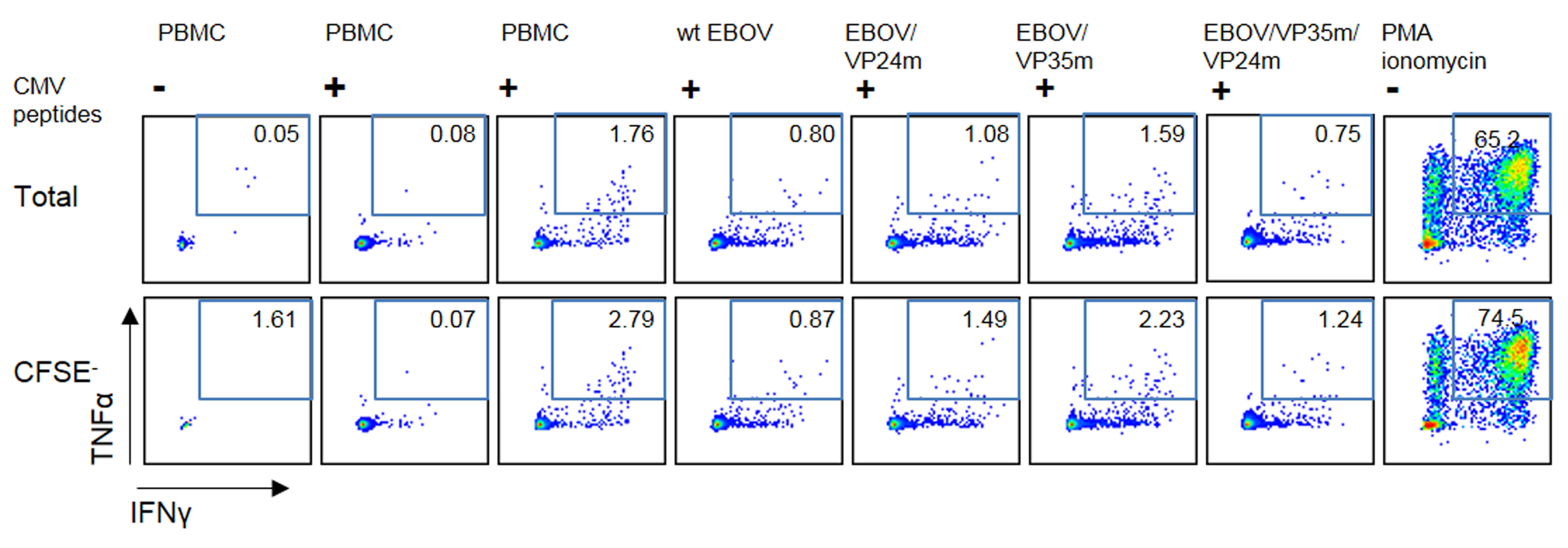

Supplement: S7 Fig — Representative primary flow cytometry data showing expression of IFNγ and TNFα by CD4+ T cells co-cultured with DCs pre-infected with the indicated viruses and simultaneously pulsed with CMV peptides gated on total CD4+ (top) and CFSE- CD4+ (bottom) T cell populations. Percentages of cells positive for the indicated cytokines are indicated in the gate. (TIF) [file ppat.1006031.s007.tif]

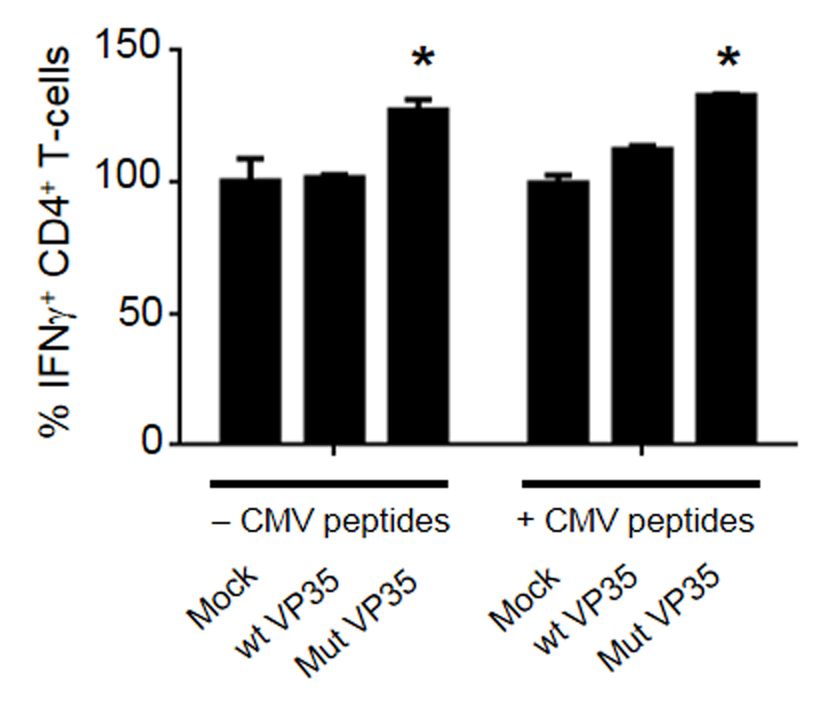

Supplement: S8 Fig — DCs were transduced with lentiviral vector expressing wt or R312A mutant VP35, incubated with or without CMV peptides, cultured with CD4+ T cells, and the percentages of IFNγ+ cells were determined by flow cytometry. Results are normalized to the mean of mock samples. Mean values with SE based on triplicate samples from one of two independent experiments performed with different donors, which resulted in essentially same results. Statistically significant differences (p<0.05) for the mutated VP35 as compared to wt VP35 are shown with asterisks. (TIF) [file ppat.1006031.s008.tif]

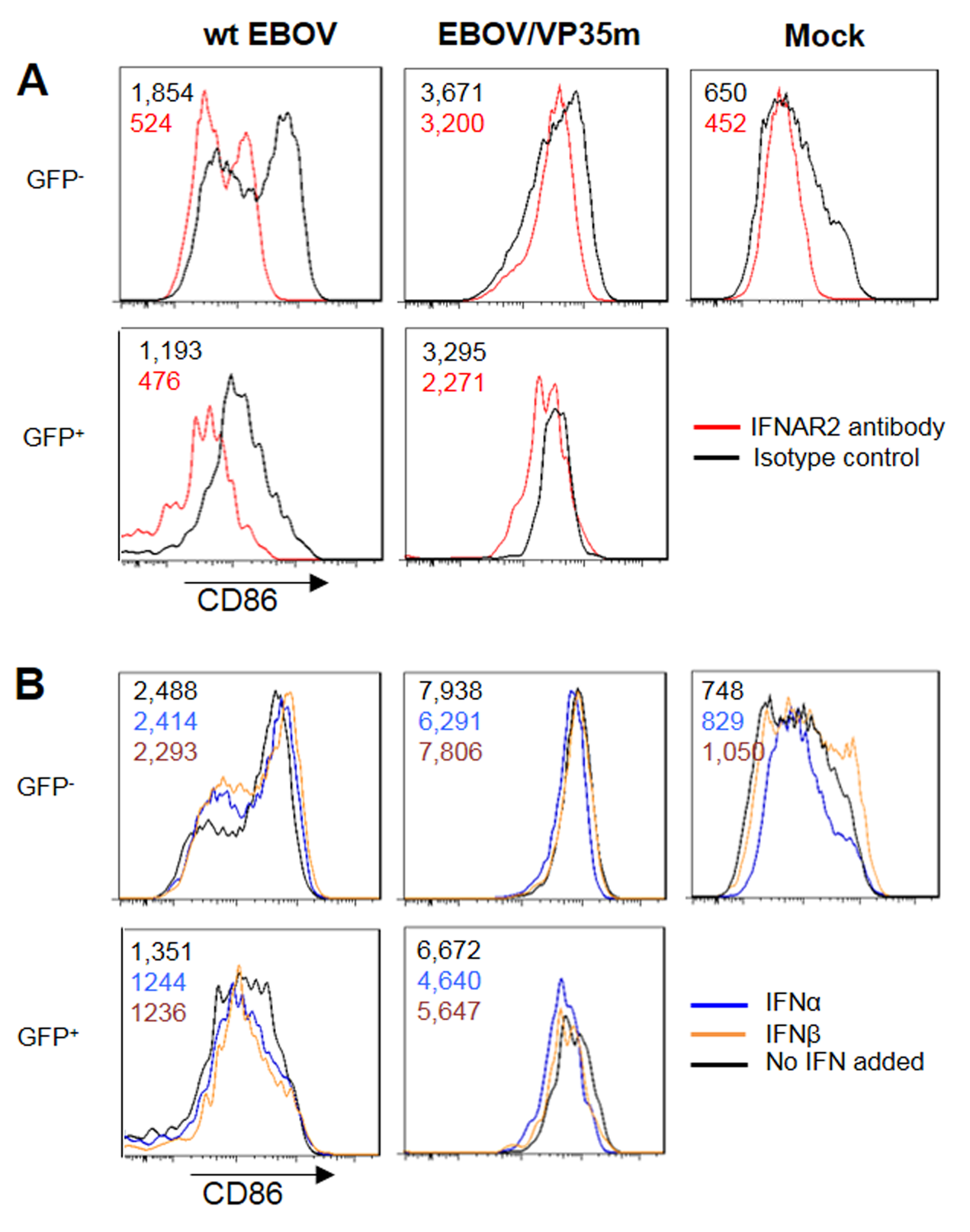

Supplement: S9 Fig — A. Effect of IFNAR2 blockade. B. Effect of exogenously added IFNα and IFNβ. MFI for CD80+ or CD54+ mock-treated DC (black), or DC treated with IFNAR2 antibodies, IFNα, or IFNβ (red, blue, brown, respectively) are indicated in upper left corners. The experiment was performed two times with essentially similar results. (TIF) [file ppat.1006031.s009.tif]

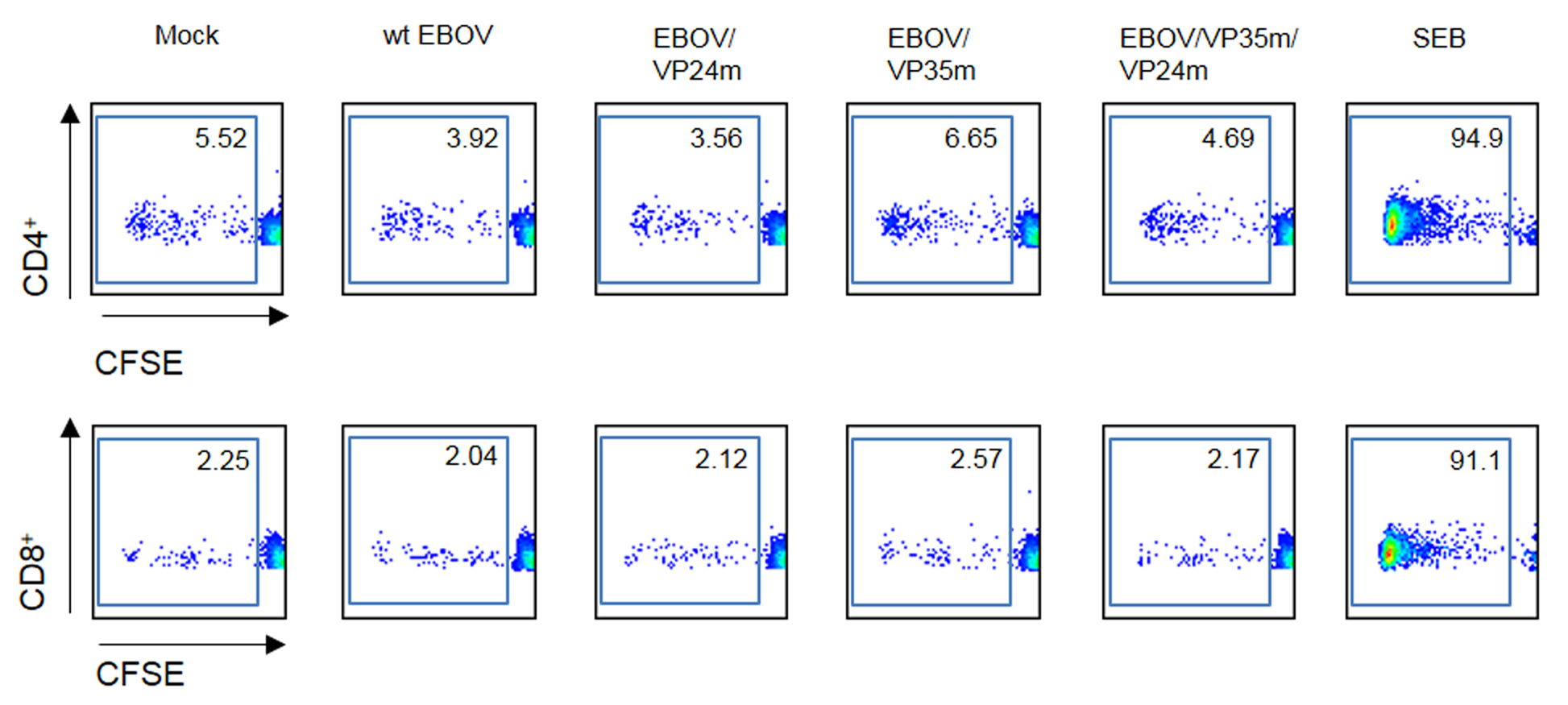

Supplement: S10 Fig — DCs were infected with wt EBOV or the mutated viruses indicated at the top and cultured with autologous CFSE-labeled CD4+ or CD8+ T cells. Proliferated CD4+ (top) and CD8+ T cells (bottom) were analyzed by CFSE dilution assay; percentages of CFSE low or negative proliferating cells are indicated. (TIF) [file ppat.1006031.s010.tif]

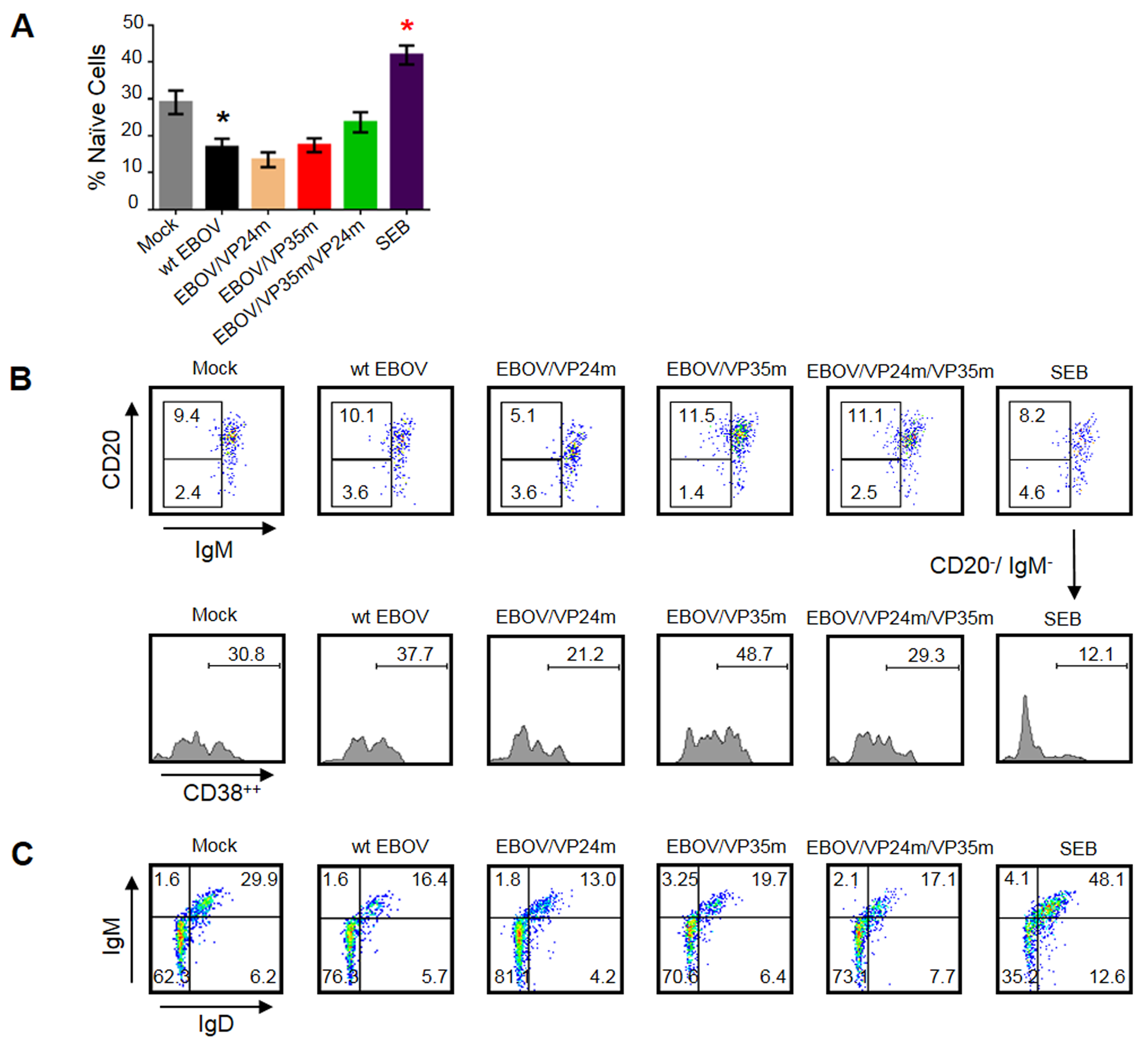

Supplement: S11 Fig — A. Percentages of naïve B cells in PBMCs infected with wt or mutated EBOVs. A statistically significant difference (P<0.05) for wt EBOV as compared to mock is indicated with the black asterisk, and for SEB as compared to wt EBOV with the red asterisk. B. Representative primary data showing gating used to analyze memory B-cell subsets shown in Fig 10B and 10C. Percentage of each gated population is shown. C. Additional subgating flow plot used to identify plasmablasts in Fig 10D. Percentages of cell populations are shown for each quadrant; percentage values used for graphs in Fig 10D in the upper right quadrant are bolded. (TIF) [file ppat.1006031.s011.tif]
