# Supplementary material for: The Ebola Interferon Inhibiting Domains Attenuate and Dysregulate Cell-Mediated Immune Responses
Source: PLoS Pathog. 2016 Dec 8;12(12):e1006031. doi: 10.1371/journal.ppat.1006031 (PMC5145241; doi:10.1371/journal.ppat.1006031)
Supplement: S1 Table — (DOC) [file ppat.1006031.s012.doc]

**Table S1. Percentages of total and proliferating (CFSE^-^) CD4^+^ T cells secreting total IFNγ, IL-2 or TNFα:** **wt EBOV values from Fig 2C**

|  | **Total** | | | **CFSE-** | | |
| --- | --- | --- | --- | --- | --- | --- |
|  | **IFNγ^+^** | **IL-2^+^** | **TNFα^+^** | **IFNγ^+^** | **IL-2^+^** | **TNFα^+^** |
| **Donor 1** | 11.9 | 0.29 | 2.59 | 15.6 | 0.37 | 3.34 |
| **Donor 2** | 0.69 | 0.45 | 0.89 | 5.56 | 5.57 | 8.34 |
| **Donor 3** | 6.69 | 0.15 | 1.83 | 16.7 | 0.49 | 6.00 |
| **Donor 4** | 8.18 | 0.2 | 5.58 | 38.4 | 1.16 | 27.9 |
| **Donor 5** | 2.01 | 0.79 | 2.50 | 11.6 | 1.65 | 8.25 |
| **Donor 6** | 12.5 | 0.12 | 7.92 | 52.8 | 0.3 | 25.7 |
| **Mean** | 7.00 | 0.33 | 3.55 | 23.44 | 1.59 | 13.26 |
| **SE** | 2.0039 | 0.1034 | 1.0836 | 7.4220 | 0.8228 | 4.3570 |
