# Supplementary material for: The Ebola Interferon Inhibiting Domains Attenuate and Dysregulate Cell-Mediated Immune Responses
Source: PLoS Pathog. 2016 Dec 8;12(12):e1006031. doi: 10.1371/journal.ppat.1006031 (PMC5145241; doi:10.1371/journal.ppat.1006031)
Supplement: S2 Table — (DOCX) [file ppat.1006031.s013.docx]

**Table S2. Percentages of total and proliferating (CFSE-) CD4^+^ T cells secreting single IFNγ^+^ or a combination of multiple cytokines: wt EBOV values from Fig. 2D**

|  | **Total** | | | **CFSE-** | | |
| --- | --- | --- | --- | --- | --- | --- |
|  | **Single IFNγ^+^** | **IFNγ^+^TNFα^+^** | **IFNγ^+^TNFα^+^IL2^+^** | **Single IFNγ^+^** | **IFNγ^+^TNFα^+^** | **IFNγ^+^TNFα^+^**  **IL2^+^** |
| **Donor 1** | 9.3 | 2.1 | 0.22 | 12.2 | 2.76 | 0.29 |
| **Donor 2** | 6.33 | 4.64 | 0.28 | 35.9 | 19.8 | 1.88 |
| **Donor 3** | 5.07 | 1.46 | 0.14 | 11.3 | 4.79 | 0.44 |
| **Donor 4** | 3.49 | 3.99 | 0.1 | 12.2 | 23.3 | 0.58 |
| **Donor 5** | 0.97 | 1.95 | 0.06 | 3.96 | 6.93 | 0.10 |
| **Donor 6** | 5.08 | 6.83 | 0.10 | 27.5 | 25.4 | 0.10 |
| **Mean** | 5.04 | 3.50 | 0.15 | 17.18 | 13.83 | 0.57 |
| **SE** | 1.1370 | 0.8397 | 0.0341 | 4.8850 | 4.1274 | 0.2741 |
