# Supplementary material for: The Ebola Interferon Inhibiting Domains Attenuate and Dysregulate Cell-Mediated Immune Responses
Source: PLoS Pathog. 2016 Dec 8;12(12):e1006031. doi: 10.1371/journal.ppat.1006031 (PMC5145241; doi:10.1371/journal.ppat.1006031)
Supplement: S3 Table — (DOCX) [file ppat.1006031.s014.docx]

**Table S3. Percentages of total and proliferating (CFSE^-^) CD4^+^ T cells secreting total IFNγ, IL-2 or TNFα: wt EBOV values from Fig. 3B**

|  | **Total** | | | **CFSE-** | | |
| --- | --- | --- | --- | --- | --- | --- |
|  | **IFNγ^+^** | **IL-2^+^** | **TNFα^+^** | **IFNγ^+^** | **IL-2^+^** | **TNFα^+^** |
| **Donor 1** | 3.62 | 0.25 | 1.02 | 5.85 | 0.38 | 1.19 |
| **Donor 2** | 1.06 | 0.04 | 0.42 | 1.22 | 0.05 | 0.48 |
| **Donor 3** | 2.36 | 0.07 | 0.64 | 3.15 | 0.09 | 0.84 |
| **Donor 4** | 27.20 | 1.25 | 2.97 | 16.60 | 2.15 | 1.13 |
| **Mean** | 8.56 | 0.40 | 1.26 | 6.71 | 0.67 | 0.91 |
| **SE** | 6.2351 | 0.2860 | 0.5819 | 3.4316 | 0.4995 | 0.1616 |
