# Supplementary material for: The Ebola Interferon Inhibiting Domains Attenuate and Dysregulate Cell-Mediated Immune Responses
Source: PLoS Pathog. 2016 Dec 8;12(12):e1006031. doi: 10.1371/journal.ppat.1006031 (PMC5145241; doi:10.1371/journal.ppat.1006031)
Supplement: S4 Table — (DOCX) [file ppat.1006031.s015.docx]

**Table S4. Percentages of total and proliferating (CFSE-) CD4^+^ T cells secreting single IFNγ^+^ or a combination of multiple cytokines: wt EBOV values from Fig. 3C**

|  | **Total** | | | **CFSE-** | | |
| --- | --- | --- | --- | --- | --- | --- |
|  | **Single IFNγ^+^** | **IFNγ^+^TNFα^+^** | **IFNγ^+^TNFα^+^IL2^+^** | **Single IFNγ^+^** | **IFNγ^+^TNFα^+^** | **IFNγ^+^TNFα^+^**  **IL2^+^** |
| **Donor 1** | 3.05 | 0.38 | 0.08 | 5.03 | 0.53 | 0.13 |
| **Donor 2** | 0.92 | 0.33 | 0.04 | 1.11 | 0.31 | 0.04 |
| **Donor 3** | 2.23 | 0.56 | 0.05 | 3.10 | 0.59 | 0.06 |
| **Donor 4** | 22.40 | 2.88 | 0.78 | 9.91 | 4.12 | 1.41 |
| **Mean** | 7.15 | 1.04 | 0.24 | 4.79 | 1.39 | 0.41 |
| **SE** | 5.1022 | 0.6161 | 0.1808 | 1.8857 | 0.9128 | 0.3335 |
