# Supplementary material for: The Ebola Interferon Inhibiting Domains Attenuate and Dysregulate Cell-Mediated Immune Responses
Source: PLoS Pathog. 2016 Dec 8;12(12):e1006031. doi: 10.1371/journal.ppat.1006031 (PMC5145241; doi:10.1371/journal.ppat.1006031)
Supplement: S5 Table — (DOCX) [file ppat.1006031.s016.docx]

**Table S5. Concentrations of cytokines and chemokines in supernatants of T cells co-cultured with DCs infected with the panel of viruses**

|  | **Mock** | **wt EBOV** | **EBOV/VP24m** | **EBOV/VP35m** | **EBOV/VP35m/**  **VP24m** |
| --- | --- | --- | --- | --- | --- |
| **CCL21** | 117.4 (7.5) | 27.9 (2.4) | 40.3 (7.4) | 99.0 (9.0) | 36.0 (2.8) |
| **CXCL13** | 1.1 (0.1) | 0.7 (0.3) | 12.6 (3.8) | 6.5 (1.3) | 27.9 (5.8) |
| **CCL27** | 5.4 (1.6) | 2.0 (0.5) | 11.3 (4.1) | 6.3 (0.9) | 10.3 (1.6) |
| **EGF** | 5.8 (0.5) | 6.4 (0.5) | 5.7 (0.4) | 6.6 (1.1) | 7.1 (0.5) |
| **CXCL5** | 3697.1 (904.6) | 458.4 (145.5) | 220.6 (26.3) | 3967.7 (875.1) | 277.7 (51.9) |
| **Eotaxin-1** | 25.5 (1.9) | 9.8 (0.7) | 8.4 (1.0) | 12.2 (0.7) | 12.3 (0.9) |
| **Eotaxin-2** | 11077.3 (1176.2) | 1195.9 (570.4) | 14119.0 (1532.7) | 14381.4 (1131.2) | 14559.4 (1590.2) |
| **Eotaxin-3** | 13.2 (1.4) | 4.3 (2.13) | 6.98 (0.6) | 8.6 (0.8) | 7.5 (0.8) |
| **FGF-2** | 75.5 (8.3) | 25.6 (3.0) | 35.7 (4.8) | 35.7 (5.8) | 41.1 (6.43) |
| **Flt-3L** | 15.9 (2.4) | 7.5 (0.9) | 8.6 (2.6) | 9.2 (3.2) | 8.2 (2.5) |
| **CX3CL1** | 190.2 (28.8) | 32.8 (2.5) | 102.3 (19.4) | 80.6 (9.5) | 81.2 (22.2) |
| **G-CSF** | 16.1 (3.7) | 3.1 (0.8) | 18.4 (2.8) | 7.9 (2.0) | 9.2 (2.7) |
| **GM-CSF** | 9.5 (0.5) | 126.4 (13.9) | 157.2 (12.0) | 51.6 (12.1) | 145.5 (13.1) |
| **GRO pan** | 1580.6 (278.9) | 301.5 (102.0) | 2061.7 (272.6) | 1598.5 (173.3) | 2182.8 (175.0) |
| **CCL1** | 2.0 (0.3) | 3.0 (1.4) | 3.7 (0.6) | 5.7 (2.8) | 15.7 (2.1) |
| **IFNα2** | 32.1 (11.5) | 63.5 (16.9) | 857.8 (368.5) | 80.8 (12.38) | 2760.8 (872.8) |
| **IFNγ** | 8.98 (2.0) | 30.6 (8.8) | 6.54 (1.5) | 46.7 (7.8) | 129.3 (38.0) |
| **IL1α** | 1.5 (0.3) | 2.8 (0.3) | 13.8 (2.6) | 3.2 (0.3) | 17.7 (5.4) |
| **IL1β** | 2.3 (0.5) | 1.5 (0.13) | 6.2 (2.1) | 6.1 (2.1) | 8.8 (3.1) |
| **IL1ra** | 3991.2 (762.9) | 1102.7 (394.7) | 1068.9 (361.1) | 2857.1 (733.7) | 1850.8 (634.4) |
| **IL2** | 26.5 (3.5) | 87.5 (7.9) | 27.3 (11.9) | 159.1 (38.9) | 150.1 (21.5) |
| **IL3** | Below | Below | Below | Below | Below |
| **IL4** | 20.6 (4.4) | 31.4 (10.4) | 37.9 (5.3) | 32.9 (4.7) | 41.2 (4.8) |
| **IL5** | 0.86 (0.3) | 8.1 (2.5) | 1.3 (0.7) | 6.9 (2.0) | 6.1 (2.9) |
| **IL6** | 150.2 (46.3) | 103.3 (21.0) | 3175.2 (439.0) | 306.3 (106.56) | 4262.2 (1376.4) |
| **IL7** | 2.9 (1.1) | 2.5 (0.2) | 3.2 (1.1) | 2.3 (1.2) | 4.0 (1.4) |
| **IL8** | 6626.3 (405.8) | 4125.6 (1087.2) | Over | Over | Over |
| **IL9** | 0.7 (0.1) | 1.7 (0.7) | 0.4 (0.1) | 1.6 (0.2) | 2.3 (0.9) |
| **IL10** | 12.9 (1.3) | 11.8 (4.1) | 6.2 (1.3) | 90.7 (38.7) | 37.7 (6.8) |
| **IL12(p40)** | 4.9 (0.9) | 4.4 (0.3) | 27.3 (4.3) | 6.2 (0.9) | 23.3 (4.2) |
| **IL12(p70)** | 12.3 (2.8) | 2.7 (1.5) | 3.1 (1.3) | 3.5 (0.7) | 2.4 (0.9) |
| **IL13** | 5.1 (1.1) | 66.4 (1.8) | 11.3 (5.7) | 201.8 (21.0) | 31.1 (9.4) |
| **IL15** | 2.9 (0.6) | 39.1 (4.5) | 40.3 (7.1) | 46.8 (4.5) | 54.0 (6.6) |
| **IL16** | 207.3 (47.6) | 208.9 (30.2) | 509.8 (45.2) | 322.6 (28.5) | 450.7 (47.2) |
| **IL17A** | 22.7 (3.1) | 3.7 (0.7) | 10.2 (1.5) | 7.9 (1.9) | 5.8 (1.5) |
| **IL18** | 10.3 (1.2) | 5.6 (0.5) | 5.1 (0.9) | 6.4 (1.6) | 13.8 (4.7) |
| **IL20** | 23.5 (6.3) | 16.4 (5.4) | 21.3 (4.7) | 34.1 (10.9) | 41.1 (12.8) |
| **IL21** | 5.0 (1.3) | 1.7 (0.5) | 2.0 (0.2) | 2.1 (0.5) | 3.5 (1.2) |
| **IL23** | 205.1 (27.3) | 89.0 (16.3) | 140.4 (20.0) | 145.3 (35.0) | 148.0 (29.6) |
| **IL28a** | 5.8 (2.8) | 4.3 (0.7) | 4.1 (1.2) | 5.9 (2.3) | 7.5 (2.0) |
| **IL33** | 5.9 (0.9) | 2.6 (0.2) | 4.9 (0.5) | 4.3 (0.8) | 3.0 (0.2) |
| **CXCL10** | 1160.4 (482.5) | 7072.8 (1477.3) | Over | Over | Over |
| **LIF** | 66.0 (13.3) | 375.3 (53.7) | 274.2 (48.1) | 239.0 (33.3) | 383.8 (54.1) |
| **MCP1** | Over | Over | Over | Over | Over |
| **MCP2** | 121.7 (27.7) | 1396.8 (656.9) | 2348.9 (240.6) | 2258.3 (393.7) | 3387.4 (482.2) |
| **MCP3** | 275.1 (47.3) | 180.2 (30.2) | 750.6 (266.7) | 8796.1 (658.7) | 5998.4 (911.3) |
| **MCP4** | 194.7 (27.3) | 80.6 (30.2) | 380.1 (27.1) | 471.6 (14.7) | 475.2 (41.9) |
| **MDC** | 20842.0 (2210.7) | 3886.2 (499.1) | 9919.5 (2790.1) | 18653.2 (1390.6) | 17662.5 (2413.0) |
| **MIP1α** | 101.3 (24.1) | 485.1 (138.2) | Over | Over | Over |
| **MIP1β** | 53.5 (16.8) | 103.1 (38.7) | 903.1 (155.8) | 394.6 (13.2) | 1645.1 (455.9) |
| **MIP1δ** | 277.6 (42.5) | 96.6 (16.0) | 369.7 (46.0) | 259.5 (33.9) | 355.4 (28.5) |
| **PDGF-AA** | 25.2 (7.6) | 82.9 (9.4) | 169.6 (16.9) | 155.1 (13.2) | 230.1 (32.1) |
| **PDGF-AB/BB** | 242.5 (21.7) | 15.3 (5.6) | 57.7 (8.7) | 85.3 (30.5) | 141.5 (31.8) |
| **RANTES** | 24.2 (6.8) | 118.5 (29.4) | 2054.4 (279.4) | 341.0 (68.5) | Over |
| **SDF1 a+b** | 341.6 (44.1) | 64.3 (10.5) | 104.6 (13.9) | 170.0 (22.5) | 144.2 (36.6) |
| **sCD40L** | 2.2 (0.3) | 2.1 (0.8) | 0.5 (0.2) | 3.0 (0.8) | 2.3 (0.5) |
| **SCF** | 5.0 (0.7) | 5.8 (1.9) | 10.5 (3.5) | 12.1 (2.6) | 11.8 (1.1) |
| **CCL17** | 325.0 (83.2) | 82.7 (27.8) | 587.7 (199.9) | 717.9 (159.8) | 705.5 (108.2) |
| **TGFα** | 68.1 (9.3) | 24.1 (5.2) | 46.1 (7.7) | 71.6 (8.4) | 76.8 (14.6) |
| **TNFα** | 37.5 (10.9) | 106.8 (26.3) | 4423.5 (1222.9) | 87.2 (16.265) | 3744.1 (220.0) |
| **TPO** | 71.2 (21.9) | 16.0 (5.7) | 36.2 (6.23) | 58.1 (5.9) | 49.1 (6.3) |
| **TRAIL** | 27.3 (5.0) | 18.9 (3.8) | 14.9 (3.2) | 27.2 (5.6) | 39.0 (4.6) |
| **TSLP** | 3.2 (0.8) | 1.1 (0.3) | 2.3 (0.3) | 2.9 (0.3) | 2.0 (0.2) |
| **VEGF** | 56.9 (4.6) | 16.7 (5.2) | 351.5 (49.5) | 39.3 (9.2) | 199.1 (32.8) |

Supernatants from CMV-pulsed DCs infected with the indicated viruses or mock-infected and cultured with expanded autologous CMV-specific T-lymphocytes were analyzed using Bio-Plex Assay 24 hours following co-culture. Average values in pg/ml ± SE based on 3 individual donors.
