# Supplementary material for: The Ebola Interferon Inhibiting Domains Attenuate and Dysregulate Cell-Mediated Immune Responses
Source: PLoS Pathog. 2016 Dec 8;12(12):e1006031. doi: 10.1371/journal.ppat.1006031 (PMC5145241; doi:10.1371/journal.ppat.1006031)
Supplement: S6 Table — (DOCX) [file ppat.1006031.s017.docx]

**Table S6. Percentages of CD4^+^ and CD8^+^ T cell proliferation by CFSE dilution assay: wt EBOV values from Fig. 7A**

|  | **CD4^+^ T cells** | | **CD8^+^ T cells** | |
| --- | --- | --- | --- | --- |
| **CMV peptides** | **-** | **+** | **-** | **+** |
| **Donor 1** | 3.4 | 6.92 | 2.04 | 3.49 |
| **Donor 2** | 3.92 | 3.68 | 1.94 | 1.86 |
| **Donor 3** | 3.74 | 10.8 | 1.47 | 5.12 |
| **Donor 4** | 2.72 | 7.75 |  | 3.87 |
| **Mean** | 3.45 | 7.29 | 1.82 | 3.59 |
| **SE** | 0.2646 | 1.4635 | 0.1757 | 0.6722 |
